# Multimodal deep learning ensemble framework for skin cancer detection

**DOI:** 10.1038/s41598-025-30534-z

**Published:** 2025-12-30

**Authors:** Mayar Ashraf Saeed, Yasmine M. Afify, Nagwa Lotfy Badr, Nivin A. Helal

**Affiliations:** 1 Bioinformatics, Faculty of Computer and Information Sciences, Ain Shams University, Cairo, 11566 Egypt; 2 Information Systems, Faculty of Computer and Information Sciences, Ain Shams University, Cairo, 11566 Egypt

**Keywords:** Skin lesion classification, CNN, Transfer learning, Ensemble learning, SMOTE, ISIC dataset

## Abstract

Skin cancer is the abnormal growth of skin cells, most often developing on skin exposed to the sun. It is among the most fatal forms of cancer, making its early detection and therapy crucial. In addition to conventional techniques, deep learning methods are increasingly utilized for accurate identification and classification. This study proposes a convolutional neural network (CNN) model using transfer learning to detect and classify multiple types of skin cancer. This study focuses on evaluating the efficacy of transfer learning techniques in enhancing CNN performance for this critical task. A significant contribution of this study is the use of transfer learning to improve CNN performance in skin cancer detection and classification by leveraging pre-trained models, including ResNet50, Xception, MobileNet, EfficientNetB0, and DenseNet121. The integration of metadata demonstrated a significant improvement in accuracy compared to using images alone, enhancing the performance of most models. Further enhancement was achieved through ensemble techniques, specifically an adaptive weighted ensemble method, which dynamically assigns weights to individual models based on their performance, resulting in superior overall accuracy. SMOTE was used as an oversampling technique to address class imbalance. The proposed fusion of pre-trained models (ResNet50, Xception, and EfficientNetB0) combined with metadata achieved 93.2% accuracy, 93% precision, 93% recall, 93% F1 score, and 97.3% AUC on the ISIC 2018 dataset. On the ISIC 2019 dataset, it achieved 91.1% accuracy, 92% precision, 93% recall, 92% F1 score, and 95.5% AUC, surpassing many state-of-the-art methods. Experiments on an external dataset, Derm7pt, resulted in 82.5% accuracy, with a precision of 86%, recall of 83%, F1 score of 84% and AUC of 89.15%, demonstrating the improved interpretability and generalization of the proposed model. The proposed ensemble model optimizes deep learning for healthcare applications, enhancing dermatological diagnosis and treatment strategies for skin cancer patients.

## Introduction

Cancer is the uncontrolled growth of abnormal cells in the body^1^. Among various forms of cancer, skin cancer has become one of the most rapidly spreading diseases worldwide. It arises when abnormal skin cells grow uncontrollably, leading to the condition known as skin cancer^2^. Early detection and accurate diagnosis are keys to successful cancer treatment.

Melanoma, the most common and deadly form of skin cancer in developed countries, is of particular concern. Other forms of skin cancer include squamous cell carcinoma^3^, basal cell carcinoma^4^, dermatofibroma^5^, Merkel cell carcinoma^6^, vascular lesions^7^, and benign keratosis^8^. Diagnostic imaging is vital for identifying abnormalities in various body parts, including the skin^9^, breast^10^, brain^10^, lung^11^, stomach cancers^12^ and colon cancer^13^. Early detection of skin cancer is crucial for better prognosis and reduced mortality rates. However, the reliability of tumor detection is often limited by insufficient sensitivity in traditional screening techniques, which are later confirmed by clinical specimens. In medical diagnostics, Artificial Intelligence (AI) is increasingly being employed by healthcare professionals to enhance and accelerate the diagnostic process. Convolutional Neural Network (CNN) architectures have shown considerable effectiveness in various medical diagnostic applications, including the detection of Parkinson’s disease through analysis of hand-drawn inputs^14^ and the classification of colon cancer using optimized MobileNetV2 models^13^. Despite some advancements, AI research in clinical diagnosis often lacks proper assessment and reporting of potential defects.

Computer-Aided Diagnosis (CAD) has proven to be an efficient and cost-effective approach for diagnosing various medical conditions, including skin cancer. Imaging techniques such as Magnetic Resonance Imaging (MRI)^5^, Positron Emission Tomography (PET)^6^, and X-rays^7^ are commonly used to assess diseases affecting human organs. In the case of skin lesions, diagnostic methods like Computed Tomography (CT) and dermatoscopy image processing are commonly employed, although their accuracy tends to decrease among less experienced dermatologists^10,11^.

The process of analysing and diagnosing skin lesions is time-consuming, difficult to standardize, and prone to errors due to the complexity of lesion imaging. Image analysis requires precise identification of lesion pixels, making it a challenging task.

The exponential growth in computational power has driven major advancements in deep learning, particularly in computer vision. CNNs have revolutionized medical image analysis, making early detection of skin cancer more attainable. Dr. Lee^15^ highlighted the increasing prevalence of skin cancer, especially among younger women, noting its early onset as one of the primary concerns. Deep learning models have outperformed human experts in several computer vision tasks^15,16^, leading to earlier diagnoses and reduced mortality rates. By integrating optimized learning strategies into deep learning models, exceptional classification and processing accuracy can be achieved^17,18^.

Despite their significant advancements, one common criticism of deep learning models is their “black box” nature, where decision-making processes are not always transparent. Nonetheless, deep learning has emerged as a powerful tool capable of classifying skin lesions with accuracy comparable to or even surpassing human specialists. The potential for improving preventive screening measures through deep learning-based programs that automatically analyse clinical and dermoscopic images is considerable.

This study introduces notable advancements in skin cancer detection by leveraging transfer learning through a set of pre-trained models, including ResNet50, Xception, MobileNet, EfficientNetB0, and DenseNet121. The integration of metadata, such as patient attributes (e.g., age, anatomical site, lesion ID, sex, and malignancy status [malignant/benign]), significantly improves accuracy when combined with image-based models. Metadata provides valuable contextual information that enhances model interpretability and allows for more precise classification by linking lesion characteristics with demographic and anatomical patterns.

An innovative ensemble technique is employed to aggregate the predictions from the highest-performing models—ResNet50, Xception, and EfficientNetB0—using an adaptive weighted method. Unlike traditional ensembles that rely on fixed or manually assigned weights, our approach introduces a dynamic weighting mechanism where ensemble weights are learned automatically during training via a dedicated trainable layer. This enables the model to optimally balance the contributions of each classifier based on their reliability and performance, effectively combining the strengths of different architectures and improving both robustness and classification accuracy.

The ensemble framework is further enhanced through the integration of a multimodal approach, combining clinical images with patient metadata (e.g., age, anatomical site, lesion ID, sex, and malignancy status). Comparative analysis between image-only and multimodal models demonstrates the clear advantage of leveraging both data types, resulting in superior diagnostic performance. To address class imbalance, we employ SMOTE (Synthetic Minority Over-sampling Technique)^19^, ensuring adequate representation of underrepresented classes and contributing to a more balanced and effective training process.

Leveraging pre-trained models enables the network to benefit from knowledge acquired from large-scale datasets, thereby reducing both training time and computational cost. This transfer learning strategy, coupled with the adaptive ensemble mechanism, significantly enhances the model’s diagnostic capability.

The proposed method shows strong performance on both the ISIC 2019 and ISIC 2018 datasets, demonstrating its ability to generalize across different distributions and surpass existing benchmarks. By integrating metadata, oversampling techniques, and a learned ensemble strategy, our approach delivers a robust and interpretable model that supports automated dermatological diagnosis and assists clinical decision-making for early skin cancer detection.

The rest of this paper consists of four main sections. Section "Related Work" examines the related work. Section "Experimental evaluation" describes the proposed model used for skin cancer detection. Section 4 shows the applied experiments along with analysis on the results. Section 5 provides conclusion and future work.

## Related work

In the previous 10 years, there has been an increase in skin cancer cases^20^. Given that the skin covers most of the body, it makes sense to think that dermatological cancer is the most frequent illness among humans. Successful treatment of dermatological cancer depends on early detection. Skin cancer signs can now be identified swiftly and simply utilizing computer-based methods. For evaluating skin cancer indicators, numerous non-invasive techniques have been suggested. Dermoscopy data was used to attempt to categorize benign and malignant skin lesions using digital image processing. Another popular method for diagnosis uses ABCD parameters of melanoma: Asymmetry—melanoma lesions generally have an asymmetrical form; Border—presence of irregular borders in melanoma lesions; Colour—melanoma lesions exhibit multiple colours; and Diameter—melanoma width is typically greater than 6 mm. The related work section in this study is structured into subsections based on the employed techniques, providing a clearer perspective on the different methodologies used for skin cancer detection.

### Transfer learning techniques

Transfer learning was applied to a deep CNN in Liao’s^21^ attempt to create a categorization for all skin diseases. The weights of the deep CNN were then fine-tuned by extending the backpropagation process. Instead of training a CNN from scratch, Kawahara et al.'s study^22^ investigated the use of a pre-trained CNN as a feature extractor for classifying non-dermoscopic skin images. It also examined how filters from a CNN trained on natural images could be repurposed to distinguish between ten different categories of non-dermoscopic skin images, demonstrating the model’s adaptability and effectiveness in feature representation.

The researchers reported new breakthrough performance in Codella et al.^23^, using ConvNets to extract picture characteristics using the Large-Scale Visual Recognition Challenge (ILSVRC) 2012 image dataset and a model that had previously been trained^24^. They also investigated the Deep Residual Network (DRN), which was the most recent network structure to triumph in the ImageNet recognition test^25^.

Sobia Bibi^26^ proposed a deep-learning architecture for classifying multiclass skin cancer and melanoma detection, consisting of four core steps: image pre-processing , feature extraction and fusion, feature selection, and classification. They introduced a novel contrast enhancement technique based on image luminance. Two pre-trained deep models, DarkNet-53 and DenseNet-201, were modified and trained through transfer learning. During learning, the Genetic Algorithm was used for hyperparameter selection. Features were fused using a two-step serial-harmonic mean approach, followed by feature selection with Marine Predator Optimization (MPA) controlled Reyni Entropy. The final classification was performed using machine learning classifiers, achieving a maximum accuracy of 85.4% on the ISIC 2018 dataset.

Nugroho et al.^27^ addressed the issue of class imbalance in the ISIC-2019 dataset by applying an extensive pre-processing and augmentation strategy using pre-trained CNNs, including Inception-V3, DenseNet-201, and Xception. Their pre-processing pipeline involved duplicate removal, metadata cleaning, image resizing, and multiple augmentation transformations such as rotation, flipping, and brightness adjustment. Using the Adam optimizer with a learning rate of 0.01 and fivefold cross-validation, the augmented dataset significantly improved model accuracy, achieving 88.63% with Inception-V3. While the study effectively demonstrated the importance of data balancing and augmentation, it relied on a single dataset and did not incorporate fine-tuning or ensemble techniques, limiting its generalizability and innovation.

Subramanian et al.^28^ presented a federated learning framework for skin cancer classification that prioritizes data privacy while maintaining high diagnostic performance across distributed datasets. The study introduced a federated architecture integrating CNN and MobileNetV2 models trained locally on four clients, two using the ISIC 2018 dataset (seven classes) and two using the ISIC 2019 dataset (eight classes). Model parameters rather than raw data were shared with a central server, where they were aggregated using the Federated Averaging (FedAvg) algorithm to form a global model. The experiments compared conventional centralized training with federated learning across multiple settings. While standalone CNN and MobileNetV2 models achieved accuracies of 83% and 89% respectively on ISIC 2018, their generalization dropped when tested on ISIC 2019. In contrast, the federated CNN attained 82% and 76% accuracy on ISIC 2018 and 2019, respectively, while the federated MobileNetV2 improved further to 80% and 87%, demonstrating stronger cross-dataset adaptability. These findings confirm that federated learning enhances generalization and privacy preservation in dermatological image analysis, addressing challenges of data centralization, domain shift, and regulatory compliance. The study highlights federated learning’s potential for real-world deployment in clinical settings where sensitive medical data cannot be shared across institutions.

### Metadata usage

Nils Gessert^29^ addressed the challenge of improving skin lesion classification by integrating patient metadata—specifically age, sex, and anatomical site—with dermoscopic image data. His model architecture processed these two data types in parallel, using a convolutional neural network (CNN) branch for dermoscopic images and a separate dense layer for metadata. The image branch included an ensemble of pre-trained architectures such as EfficientNet variants, SENet154, and ResNeXt models, chosen for their high performance in image recognition tasks. The metadata branch contributed contextual clinical information that could assist in distinguishing between lesions with similar visual features. The model was evaluated using five-fold cross-validation on the ISIC 2019 dataset and achieved a balanced accuracy of 74.2%. Incorporating metadata led to slight improvements in performance, particularly for smaller models, although the benefit was less pronounced when applied to the official test set. Overall, the study demonstrated that integrating patient metadata with image-based models can provide marginal gains in classification accuracy and improve robustness in some scenarios.

Qilin Sun^30^ used the ISIC 2019 dataset along with additional images and applied image pre-processing techniques like Shades of Gray colour constancy. Metadata was encoded using one-hot encoding for anatomical site and age, while sex was represented numerically. His model architecture was based on EfficientNet (B3 & B4), integrating a dense neural network for metadata fusion. To enhance performance, geometric and pixel-wise data augmentation was applied, and Test Time Augmentation (TTA) was used during inference. His model was trained for 60 epochs with SGD and OneCycle learning rate scheduling, utilizing weighted cross-entropy loss, which outperformed focal loss. His results showed 88.7% accuracy for a single model and 89.5% for an ensemble model on the ISIC 2018 test set, making it top-ranked on the ISIC leaderboard. Similarly, on ISIC 2019, his ensemble model achieved 66.2% accuracy. Additionally, Grad-CAM visualization helped highlight critical regions for diagnosis, assisting clinicians. His findings demonstrated that integrating patient metadata and TTA significantly improved classification accuracy while maintaining computational efficiency, making it practical for real-world use.

Yali Nie^31^ used clinical patient metadata, including age, sex, lesion location, and clinical history, to enhance skin cancer classification on the ISIC 2018 dataset. His research conducted six experiments with different model architectures, including CNN-based models, Vision Transformers (ViT), and hybrid CNN-ViT models. For image pre-processing , he applied extensive data augmentation techniques, including random flipping, rotation, brightness adjustment, and colour jittering, to improve model generalization and reduce overfitting. To address class imbalance, he employed the focal loss (FL) function, which significantly improved classification performance compared to standard cross-entropy loss. On the metadata pre-processing side, he handled missing values in the clinical data, normalized numerical attributes like age, and balanced the metadata distribution to avoid model bias towards certain demographic groups. Unlike other research that combined image data and metadata for classification, his research concentrated solely on image-based deep learning techniques. His best-performing model, a hybrid CNN-ViT model with FL, achieved an accuracy of 89.48%, surpassing prior state-of-the-art methods. His evaluation metrics like AUC, F1 score, precision, and recall showed significant improvements, especially for underrepresented classes such as actinic keratosis (AKIEC) and vascular lesions (VASC). His research highlighted the importance of using advanced image-based deep learning techniques like hybrid CNN-ViT models while suggesting that integrating patient metadata could further improve classification performance and diagnostic accuracy in dermatology applications.

Wenjun Yin^32^ focused on improving skin tumor classification by integrating clinical patient metadata—such as age, sex, and medical history—with image data through a deep convolutional network. His research proposed a novel architecture that combined the MetaNet and MetaBlock modules with the well-established DenseNet-169 network, known for its efficient feature propagation and reuse. By incorporating clinical metadata, his model enhanced its ability to make more informed decisions based on both visual patterns and patient-specific information. The MetaNet and MetaBlock modules effectively fused image and metadata features, enabling his network to learn from both the detailed visual characteristics of skin lesions and the contextual medical data, ultimately improving performance. Evaluated on the ISIC 2019 dataset, his proposed model achieved a balanced accuracy of 81.4%, demonstrating a significant improvement over previous method. This enhancement, ranging from 8% to 15.6% compared to earlier image-only classification models, highlighted the impact of integrating patient metadata. His research emphasized the importance of combining clinical patient data with deep learning models to enhance the precision of skin cancer diagnosis, showing that leveraging both clinical context and image data resulted in a more robust and reliable diagnostic tool for dermatological applications.

### Ensemble techniques

Ensemble techniques in machine learning are used to combine multiple models, often previously trained models, to improve performance. This approach is frequently employed to provide more precise and dependable outcomes. It’s applied in various studies as:

Ahmet DEMR^33^ created a useful technique for early skin cancer diagnosis. His dataset included 2,437 practice images. Challenges involved in classification were solved using a variety of deep learning systems. After data analysis, the response score for the Inception v3 design was 87.42%, while the response score for the ResNet 101 design was 84.09%.

By using AI-augmented detection techniques, Subhranil Bagchi^34^ aimed to accomplish this goal at a lower cost and in less time than with traditional approaches. His research improved accuracy over individual classification models by using a two-level ensemble learning strategy (trained with weighted losses). By reducing overfitting caused by the dataset’s class imbalance, the ensemble approach achieved a Balanced Multi-class Accuracy (BMA) score of 59.1% without the need for unknown class identification. To detect the existence of photos belonging to novel classes during test time, the proposed CS-KSU module collection was appended to the method. For the unidentified class, the enhanced method achieved an Area Under the ROC Curve (AUC) score of 0.544.

Josef Steppan^35^ assessed the state-of-the-art in dermoscopic image classification using the most recent research and the ISIC 2019 Challenge for skin lesion classification. He applied various models using the transfer learning technique to classify eight classes of skin lesions. Input data was randomly altered based on predetermined criteria (translation, rotation, scaling, etc.) during training. Cutout was also applied for regularization. For training, a total of 32,748 images were available. To create training data, only images from SD-198 were utilized. The “UNK” class was introduced after eliminating image data from the eight classes in the training dataset for ISIC-2019. Various models were applied, such as EfficientNet-B5, SE-ResNeXt-101(32 × 4d), EfficientNet-B4, Inception-ResNet-v2, and NASNet-A-Large, which achieved accuracies of 60%, 58.2%, 57.7%, 56.9%, and 50.4%, respectively. Then, he applied the ensemble technique (excluding NASNet) to these pre-trained models, achieving an accuracy of 63.4%.

Cauvery^36^ applied an online augmentation strategy to address the issue of unbalanced classes. His method’s need for an internet connection, increased processing cost, reliance on input data quality, and potential for overfitting outweighed its advantages, which included not directly increasing the number of training images. He aimed to develop a model to classify the eight classes of the ISIC 2019 challenge dataset and applied an ensemble technique integrating DenseNet-V2, Inception-V3, InceptionResNetV2, and Xception to effectively combine predictions generated by the sub-models. He used the Adam optimizer with an initial learning rate of 1e-3 and trained the model for 50 epochs (starting from the fourth epoch) with a batch size of 64. His ensemble achieved an accuracy of 82.1%.

Sekineh Asadi Amiri^37^ introduced an ensemble model that integrated Inception-ResNet v2 with a Soft-Attention mechanism and an optimized EfficientNet-B4. His model achieved superior performance on the ISIC-2017 and ISIC-2018 datasets. By employing soft voting, an accuracy of 88.21% was achieved on the ISIC-2018 dataset, surpassing the results of individual models and previous state-of-the-art approaches. This improvement demonstrated the effectiveness of combining multiple architectures to leverage their complementary strengths. Various image augmentation techniques, such as rotation, zooming, shifting, and reflection, were applied. During pre-processing , nearest-neighbor interpolation resized images to 299 × 299 pixels for Inception-ResNet v2 and 380 × 380 pixels for EfficientNet-B4. The Soft-Attention mechanism in Inception-ResNet v2 enhanced feature extraction by focusing on informative lesion regions while suppressing noise, whereas the additional dense layers in EfficientNet-B4 contributed to improved classification performance. Through this ensemble approach, both accuracy and model robustness were enhanced, highlighting its potential for real-world melanoma detection applications.

S. Talayeh Tabibi^38^ proposed an ensemble classifier for skin lesion classification using multiple Convolutional Neural Networks (CNNs). Her research focused on increasing diversity at both the data and classifier levels to enhance model robustness and accuracy. To achieve this, bootstrapping was applied to generate varied training subsets, and Cohen’s Kappa score was used to eliminate highly correlated models, ensuring better ensemble diversity. The dataset used was ISIC 2018, containing over 13,000 dermoscopic images across seven classes. Different CNN architectures were experimented with, including ConvNext, SENet, DenseNet, and EfficientNet, selecting the best-performing models based on accuracy and diversity. The final ensemble classifier, utilizing a majority voting strategy, combined ConvNext-Tiny, EfficientNetB0, SENet, DenseNet, and ResNet50 to enhance classification performance. Additionally, various pre-processing techniques were applied, including data augmentation, normalization, and resizing images to 240 × 240 pixels. This comprehensive approach contributed to her model’s robustness, leading to a final accuracy of 90.15%, surpassing individual models and many existing methods in skin lesion classification.

In recent studies, H. Fırat^39^ proposed ensemble and attention-based deep learning frameworks have gained prominence for skin lesion classification. For instance, DXDSENet-CM (2024) introduced an ensemble model combining Xception, DenseNet201, and a Depthwise Squeeze-and-Excitation ConvMixer (DSENet-ConvMixer) to improve multi-class lesion detection. The proposed approach integrated the feature extraction capabilities of pre-trained convolutional backbones with depthwise attention mechanisms to enhance both global and local representation learning. The ensemble framework aggregated predictions from the three models, demonstrating improved robustness compared to individual architectures. Experiments conducted on the ISIC 2018 dataset showed that the ensemble significantly outperformed single networks, achieving an accuracy of 88.21%. These results highlight the effectiveness of leveraging complementary deep models and channel attention modules to improve classification generalization and stability across diverse dermoscopic image distributions.

### Custom CNN model

Pandey et al.^40^ developed a deep learning framework for skin cancer classification using the ISIC-2019 dataset, combining Non-Local Means (NLM) denoising, Sparse Dictionary Learning, and a CNN model to enhance image quality and classification accuracy. The pre-processing involved resizing images to 100 × 100,100 \times 100,100 × 100, applying NLM denoising, performing rotations and flips for data augmentation, and using class weighting to address class imbalance. Sparse Dictionary Learning (64 atoms, α = 1, 100 iterations) was applied to improve feature representation before CNN training. The CNN employed a bottleneck architecture with filters (128, 256, 512, 512, 256), ReLU and Softmax activations, and Adam optimization with batch normalization. The model achieved 81.23% accuracy on the ISIC-2019 dataset, demonstrating that combining denoising and sparse feature learning can effectively improve CNN performance. However, the study did not incorporate transfer learning, fine-tuning, or ensemble techniques, which could potentially enhance model generalization and further improve classification performance.

### Summary

Comprehensive analysis of the related works highlighted the great impact of the use of augmentation and pre-processing techniques which increased the amount of training data. They increase the model’s ability to generalize, add variability to the data and minimize data overfitting, save on the cost of collecting and labelling additional data, and ultimately improve the accuracy of the deep learning model’s predictions.

While many studies have explored deep learning for skin cancer classification, several limitations persist. Most evaluate models only on benchmark datasets like ISIC 2018 and 2019, lacking external or cross-dataset validation, which raises concerns about generalizability to diverse clinical environments. Additionally, complex pre-processing pipelines and handcrafted features increase computational overhead, limiting real-time feasibility. Limited comparisons with standard end-to-end architectures and the absence of interpretability tools like Grad-CAM further restrict clinical trust and adoption.

This work addresses these gaps by introducing an adaptive weighted ensemble technique that dynamically learns optimal model contributions, improving robustness and accuracy beyond fixed-weight methods. By integrating multimodal data—including clinical images and patient metadata such as age, anatomical site, sex, lesion features, and malignancy status—and employing SMOTE to balance underrepresented classes, our approach enhances diagnostic performance. Grad-CAM visualizations improve interpretability, supporting clinical relevance. Extensive external cross-dataset evaluations demonstrate strong generalizability and scalability for practical melanoma detection.

## Proposed model

This study presents a robust model for skin cancer detection utilizing five pre-trained models— ResNet50, Xception, MobileNet, EfficientNetB0, and DenseNet121—through transfer learning. A key innovation of this approach is the integration of metadata, such as patient demographics and lesion characteristics, with high-dimensional image features. This dual-input strategy was rigorously evaluated on two datasets, ISIC 2018 and ISIC 2019, yielding substantial improvements in model accuracy by incorporating contextual data alongside image-based features.

The metadata, comprising attributes like age, anatomical site, and sex, is seamlessly concatenated with the deep features extracted by the pre-trained models. This fusion enables the models to leverage both visual and contextual information, facilitating a more comprehensive understanding of the input data. Consequently, the model’s ability to discern subtle variations and patterns in skin lesions is significantly enhanced.

To further improve the classification performance, an ensemble technique is employed, utilizing the top-performing models— ResNet50, Xception, and EfficientNetB0. This ensemble approach capitalizes on the strengths of each model, combining their outputs to reduce prediction variance, mitigate individual biases, and increase diagnostic reliability. The choice of these three models is motivated by their complementary strengths: EfficientNetB0 offers scalable accuracy through compound scaling, Xception excels in capturing fine-grained details with depthwise separable convolutions, and ResNet50 leverages residual connections to enable deeper architectures and more hierarchical feature extraction. By integrating these models, the ensemble approach ensures a more stable and balanced decision-making process.

The selection of three models— ResNet50, Xception, and EfficientNetB0—is strategically made to balance diversity in learned features while maintaining high performance and computational efficiency. This configuration maximizes feature extraction capabilities and enables a well-rounded classification system that is robust across a variety of lesion types.

Additionally, the incorporation of structured metadata alongside image-derived deep features enriches the model’s contextual understanding, leading to superior classification performance. This integrated approach significantly enhances the system’s ability to capture complex patterns in skin lesions, outperforming traditional two-model ensembles in both accuracy and reliability. The datasets, pre-processing techniques, and the proposed model architecture are presented in the following subsections.

### Datasets

The ISIC 2018^41^ and ISIC 2019^42^ datasets were selected for this study due to their comprehensiveness, high quality, and relevance to the task of skin cancer detection^27,28,37–40^. These datasets are part of the largest publicly available collections of annotated dermoscopic images, specifically curated for research in melanoma and skin lesion classification. The ISIC 2018 dataset includes a diverse range of lesion types and is benchmarked for tasks such as lesion segmentation and disease classification, providing a robust foundation for developing and evaluating deep learning models. The ISIC 2019 dataset, which expands upon this, offers an even larger and more varied collection of images across multiple classes, including rare skin cancer types, allowing for more granular model evaluation. Together, these datasets present real-world variability in skin lesions, ensuring that models trained on them are well-equipped to generalize and perform effectively in clinical settings. Their extensive use in the research community also facilitates direct comparison with existing methods, making them ideal for demonstrating the effectiveness of novel approaches. Particularly for the automatic identification and categorization of skin lesions, both datasets are critical to the advancement of dermatological machine learning algorithms.

#### ISIC 2018 dataset

**Fig. 1:**
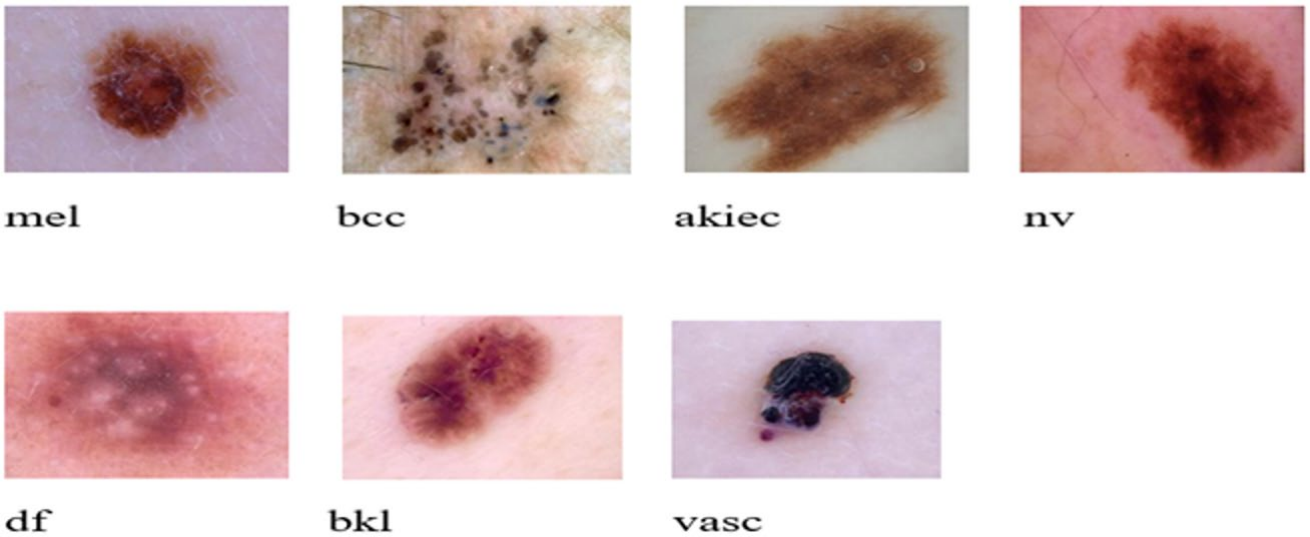
ISIC 2018 dataset samples.

**Fig. 2:**
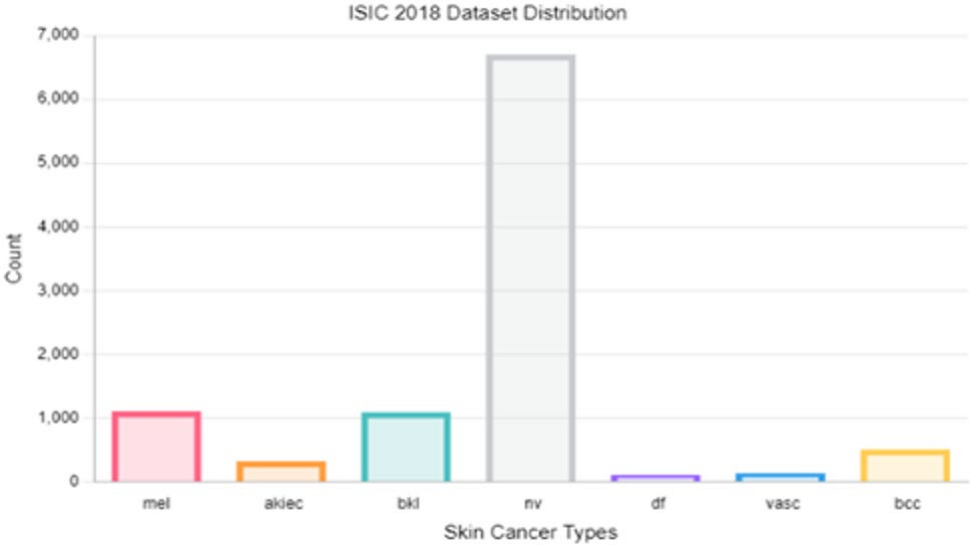
Samples distribution in the ISIC 2018 dataset.

The International Skin Imaging Collaboration (ISIC) 2018 Challenge Dataset^41^ stands as a pivotal resource in the realm of dermatology and medical image analysis. The ISIC 2018 was used as the source for both the training and testing datasets for this study. The ISIC 2018 challenge dataset comprises approximately 10,015 dermoscopic images. The training dataset comprises of 31.181 sample points, with a range of pixel counts for each image. Every sample point is categorized into one of the seven types of skin which are: Basal Cell Carcinoma (BCC), Benign Keratosis-Like Lesions (BKL), Melanocytic Nevi (NV), Dermatofibroma (DF), Melanoma (MEL), Vascular Lesion (VASC), Actinic Keratosis (AKIEC), each image is meticulously annotated with valuable metadata, offering a comprehensive context for research and analysis. Figure 1 displays a few of the example pictures. Additionally, Fig. 2 shows the distribution and often each of these seven kinds appeared in the training dataset (ISIC 2018 Dataset). The final dataset consists of 7 classes, of which samples are displayed in Fig. 1.

#### ISIC 2019 dataset

**Fig. 3:**
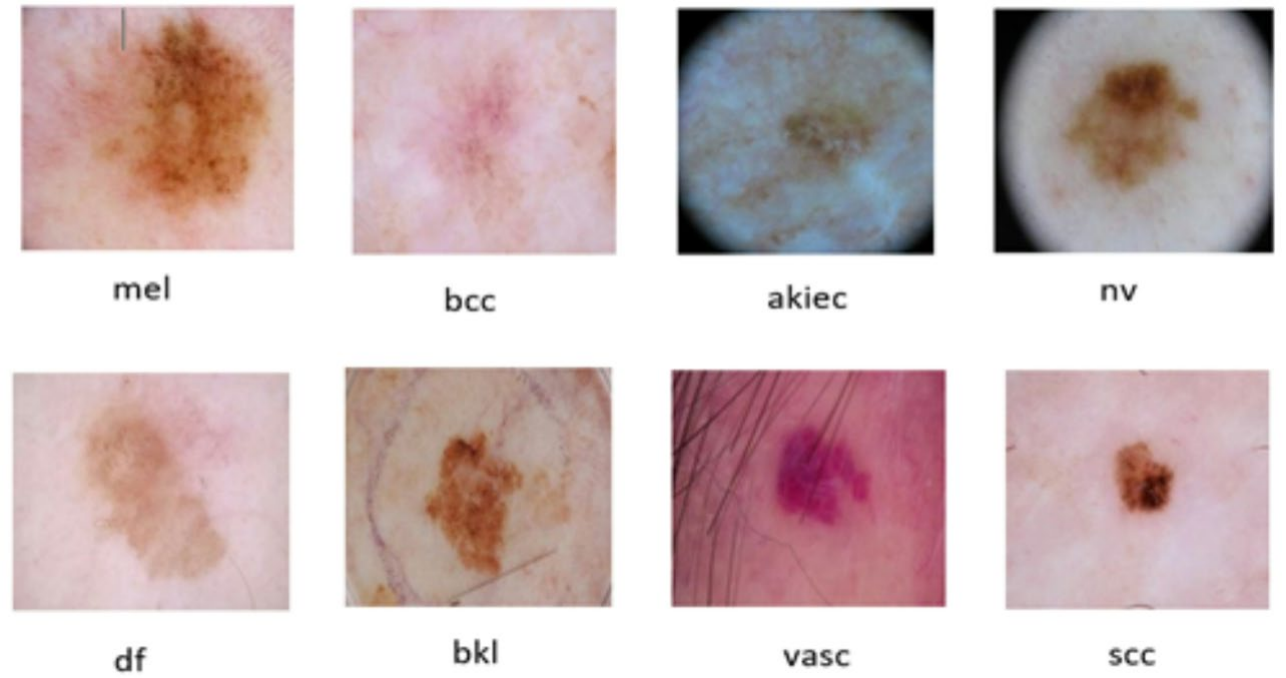
ISIC 2019 dataset samples.

**Fig. 4:**
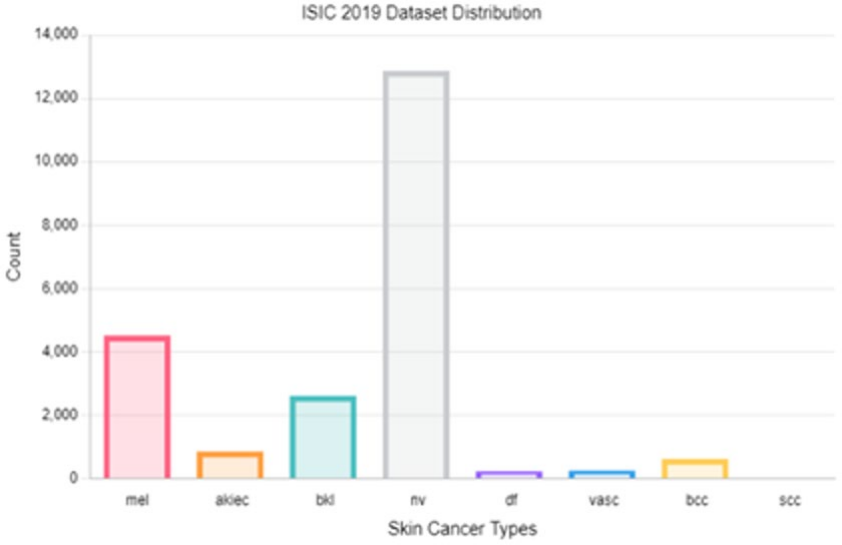
Samples distribution in the ISIC 2019 dataset.

Similarly, the ISIC 2019 Challenge Dataset^42^ extends the legacy of its predecessor, building upon the success and impact of the ISIC initiative. This dataset continues to push the boundaries of dermatological research by providing an extensive collection of skin images with diverse lesions and conditions. The ISIC 2019 dataset, similar to its precursor, includes detailed annotations and clinical information for each image, enabling researchers to delve deeper into the complexities of skin pathology, and it’s composed of 25,331 dermoscopic images. It includes 8 types of skin cancer which are: Basal Cell Carcinoma (BCC), Benign Keratosis-Like Lesions (BKL), Melanocytic Nevi (NV), Dermatofibroma (DF), Melanoma (MEL), Vascular Lesion (VASC), Actinic Keratosis (AKIEC), and squamous cell carcinoma (SCC). The distribution and frequency of each of these eight types in the training dataset (ISIC 2019 Dataset) are displayed in Fig. 3. Eight classes make up the final dataset. The distribution and frequency of each of these eight types in the training dataset (ISIC 2019 Dataset) are displayed in Fig. 4. Table 1 compares between the two datasets.

**Table 1:** Comparison between ISIC 2018 and ISIC 2019 datasets.

Feature	ISIC 2018	ISIC 2019
Challenge year	2018	2019
Total images	Approximately 10,000	Approximately 25,000
Image resolution	Varies	
Primary task(s)	Lesion segmentation	Multi-class lesion classification
	Lesion attribute detection	
	Disease classification	
Number of classes	7 classes	8 classes
Classes	Melanoma	Melanoma
	Melanocytic nevus	Melanocytic nevus
	Basal cell carcinoma	Basal cell carcinoma
	Actinic keratosis	Actinic keratosis/Bowen’s disease (intraepithelial carcinoma)
	Benign keratosis (solar lentigo/seborrheic keratosis/lichen planus-like keratosis)	Benign keratosis-like lesions
	Dermatofibroma	Dermatofibroma
	Vascular lesion	Vascular lesions
		Squamous cell carcinoma
Annotations provided	Lesion boundaries (segmentation masks)	Image-level labels
	Lesion attributes (e.g., globules, streaks)	
Evaluation metrics	Dice coefficient for segmentation	Balanced multiclass accuracy
	ROC-AUC for classification	
Data source	Multiple clinical centres worldwide	
Ground truth	Expert dermatologists’ annotations	
Availability	Publicly available for research purposes	
Gaps and challenges	Class Imbalance	
	Quality and variability of annotations	Complexity of multi-class classification
	Limited number of images	Noise in labels
	Single modality	Intra-class variability
	Lack of longitudinal data	Limited contextual information
	Common challenges	
	Data augmentation and generalization	
	Evaluation metrics	
	Interpretability of models	
	Integration into clinical workflows	
	Ethical and privacy concerns	

#### Derm7pt dataset

The Derm7pt^43^ dataset is a publicly available dermatological image dataset designed to support the development and evaluation of automated skin lesion diagnosis systems. It contains over 2,000 images, including both clinical and dermoscopic views, annotated with diagnostic labels as well as the seven-point checklist criteria—clinically relevant features used by dermatologists to assess skin lesions, such as atypical pigment networks, blue-white veil, and irregular streaks. This inclusion of intermediate semantic attributes enables more interpretable model predictions and facilitates multi-task learning, where models can be trained to predict both diagnosis and associated visual features. The dataset includes a diverse range of diagnostic classes, such as MEL (melanoma), NV (nevus), BKL (benign keratosis-like lesions), DF (dermatofibroma), VASC (vascular lesions), and BCC (basal cell carcinoma). Many of these classes overlap with those in the ISIC 2018 Challenge dataset, making Derm7pt a complementary resource for benchmarking skin lesion classifiers. However, Derm7pt lacks the AKIEC (actinic keratosis and intraepithelial carcinoma) class, which is present in ISIC 2018.

**Fig. 5:**
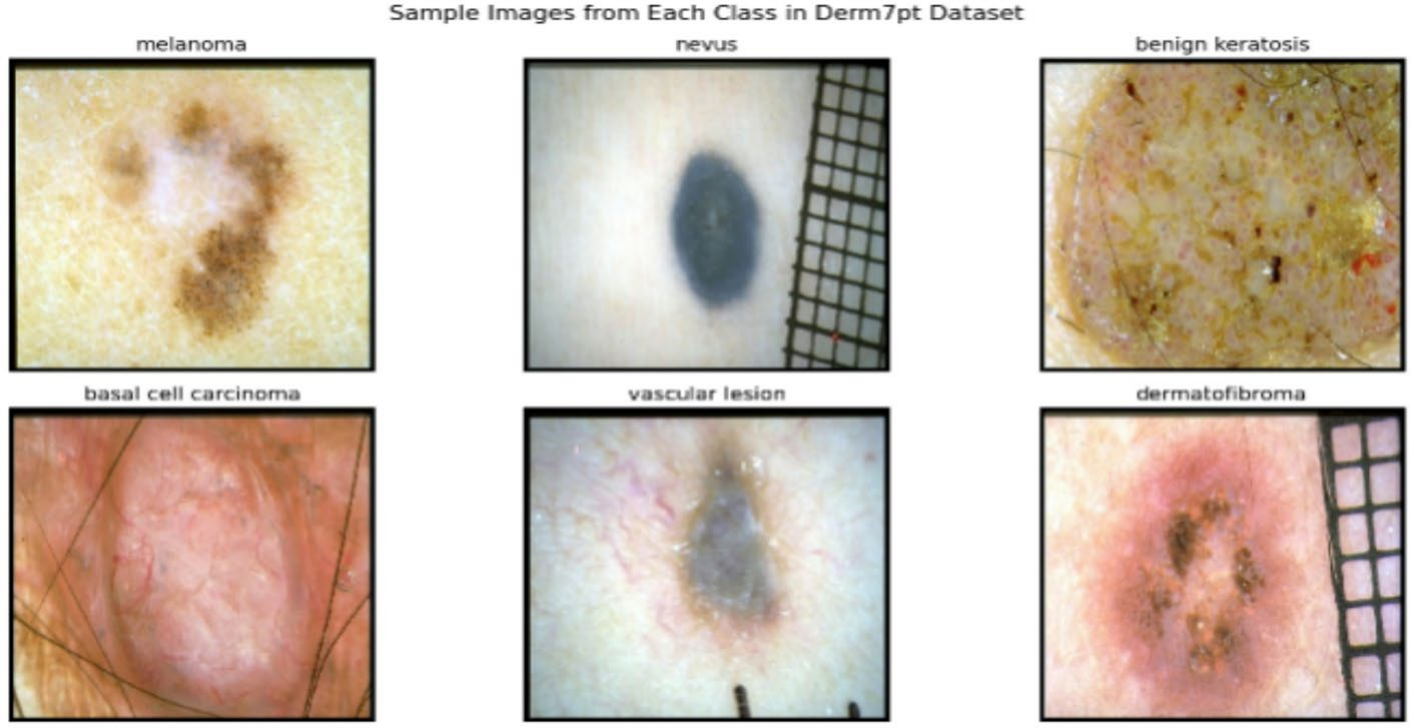
Sample images from the Derm7pt dataset showing common classes.

Despite some class imbalance issues, Derm7pt’s rich annotations and multimodal images make it valuable for developing interpretable and clinically relevant deep learning models. Table 2 shows the distribution of the common classes by number. Figure 5 shows sample of each class of these common classes in Derm7pt dataset.

**Table 2:** Class Distribution in the Derm7pt Dataset.

Class name	Abbreviation	Number of images
Melanocytic Nevi	NV	575
Melanoma	MEL	251
Benign Keratosis-like Lesions	BKL	45
Basal cell carcinoma	BCC	42
Vascular lesions	VASC	29
Dermatofibroma	DF	20
Total	–	962

### Pre-processing techniques

Pre-processing is a crucial step to refine data quality and enhance model performance. This section is divided into two subsections: pre-processing for images and pre-processing for metadata. Each focus on preparing the respective data types to ensure they are properly structured and optimized for training.

#### Image pre-processing

Pre-processing techniques are vital in skin image analysis, managing challenges like image variability. They ensure uniformity for effective model training, while addressing issues such as annotation consistency and class imbalance. Challenges persist in adapting models for clinical use, driving ongoing research and collaboration in dermatological diagnostics. In this study, the pre-processing phase focuses primarily on two key steps: image resizing and data augmentation, which are described in the following paragraphs.

##### Data augmentation

Data augmentation is a crucial technique to address the class imbalance in both the ISIC 2018 and ISIC 2019 datasets, which are commonly used for skin cancer detection. By generating synthetic variations of existing images, data augmentation helps increase the diversity of underrepresented classes, thus mitigating the risk of model bias toward the more prevalent classes. In this study, various augmentation techniques were applied with specific parameter values to enhance dataset diversity and model performance. These techniques include random rotations with a rotation range of 30, flips, translations with a width shift range of 0.3 and a height shift range of 0.3, shear with a shear range of 0.3, zooming with a zoom range of 0.3, and adjustments to brightness and contrast, which preserve the essential features of skin lesions while enhancing the dataset’s size. Techniques like horizontal flipping being set to true and applying a nearest neighbour fill mode ensure a more balanced learning process and improved performance, particularly for rare skin lesion types.

##### Resize technique

ISIC 2018 and ISIC 2019 datasets consist of images of different sizes and dimensions while convolution neural networks require images of identical sizes to work properly. For this reason, resizing images is essential as a pre-processing step. All images in the two sets were normalized and resized to 224 × 224 pixels through the ‘ImageDataGenerator’ from Keras^44^.

#### Metadata pre-processing

Metadata pre-processing plays a crucial role in enhancing model performance by ensuring the quality and consistency of non-image features. It addresses common issues such as missing values and class imbalance, which can negatively impact learning and generalization. Techniques like missing value imputation and oversampling are applied to create a more balanced and complete dataset. In this study, the pre-processing phase focuses primarily on two key steps: handling missing values feature encoding, feature scaling, and applying oversampling, which are described in the following paragraphs.

#### Metadata features

In this study, a subset of clinical metadata features was selected based on domain knowledge and relevance to the classification task. The chosen features included: age_approx (patient’s approximate age), anatom_site_general (general anatomical site of the lesion), benign_malignant (lesion malignancy status), sex (patient’s sex), and diagnosis (disease category).

Prior to modeling, the distributions of the metadata were analyzed to understand the data characteristics and detect potential imbalances. Visualizing these distributions guided the application of data preprocessing techniques such as imputation for missing values and one-hot encoding for categorical variables, ensuring the model receives clean and informative input.

**Fig. 6:**
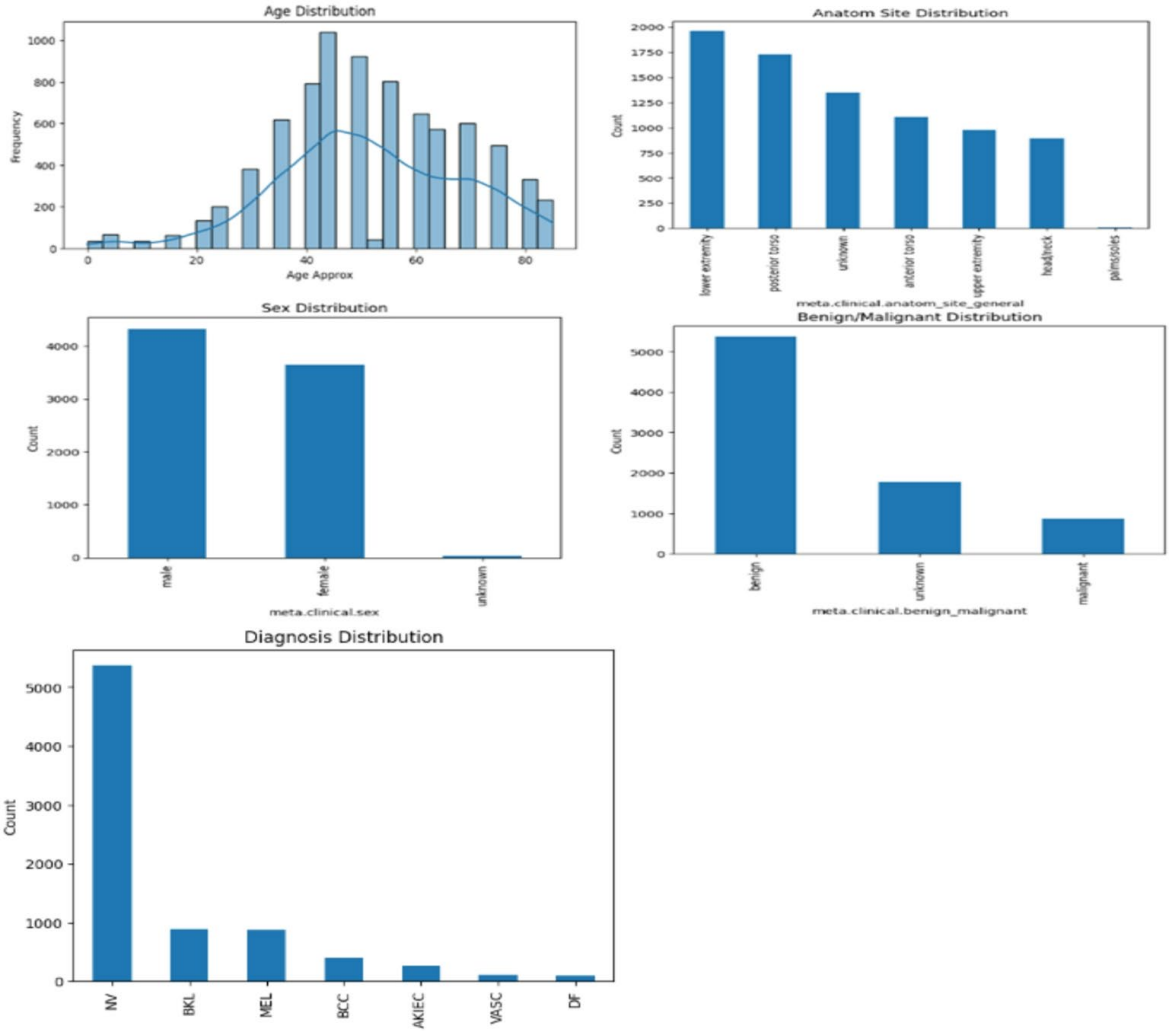
Metadata features distribution of ISIC 2018 dataset.

**Fig. 7:**
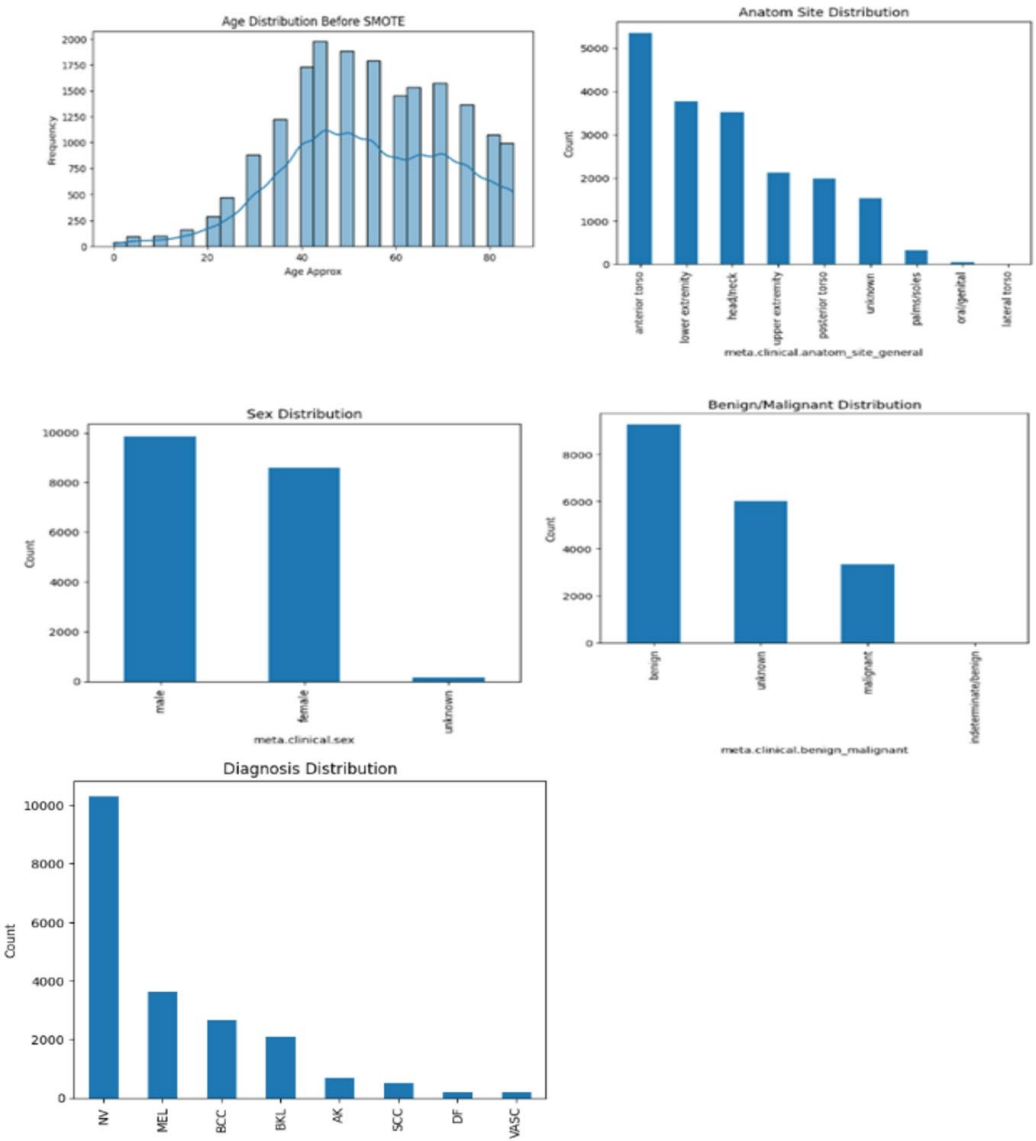
Metadata features distribution of ISIC 2019 dataset.

Figures 6 and 7 show the distribution of metadata features of ISIC 2018 and ISIC 2019 datasets respectively.

##### Handling missing values

To effectively handle the missing data in our dataset, we identified missing values in the ‘age_approx’ column and imputed them using the mean age across the dataset. For categorical columns with missing values, such as ‘sex’ and ‘anatom_site_general’, we replaced the missing entries with a default value of ‘unknown’. Rather than excluding these rows, this imputation approach allowed us to maintain the integrity and completeness of our data while ensuring that our model could still leverage valuable information from entries with previously missing data. After this pre-processing step, we proceeded to scale the numerical features and encode the categorical ones, allowing us to seamlessly integrate both metadata and image data into our model for more accurate predictions. This method ensured a balanced treatment of missing data while preserving the overall quality and quantity of the dataset.

##### Feature encoding

To prepare the categorical metadata for input into the machine learning model, feature encoding was applied using One-Hot Encoding. The categorical variables selected for encoding were meta.clinical.sex, meta.clinical.anatom_site_general, and meta.clinical.benign_malignant. Using Scikit-learn’s OneHotEncoder with sparse_output = False, each unique category within these columns was transformed into a separate binary column. This process ensures that the model does not assume any ordinal relationship between categories and treats each class independently. For example, the meta.clinical.sex column, which includes values such as “male,” “female,” and “unknown,” was expanded into three distinct binary features. Even binary columns like meta.clinical.benign_malignant were encoded into two separate columns to maintain consistency across all categorical variables. This encoding step is essential for enabling the model to effectively interpret categorical data and learn meaningful patterns without introducing bias from the feature representation.

##### Feature scaling

Feature scaling is an essential preprocessing step in machine learning, particularly when numerical features have different ranges or units. In this project, the numerical metadata feature meta.clinical.age_approx was standardized using Scikit-learn’s StandardScaler. This method transforms the data so that it has a mean of zero and a standard deviation of one. Standardizing the age feature ensures that it contributes appropriately during model training and prevents it from having an outsized influence due to its original scale. This step helps the model learn more effectively and improves the overall stability and performance of the training process.

##### Oversampling

Oversampling is a critical technique employed in machine learning and data analysis to address class imbalance within datasets. Class imbalance arises when certain classes have significantly fewer instances compared to others, leading to biased model performance that favours the majority class. To mitigate this issue, oversampling methods artificially increase the number of samples in the minority classes, thereby achieving a more balanced dataset.

One of the most widely used techniques for addressing class imbalance is the Synthetic Minority Oversampling Technique (SMOTE)^19^, which generates synthetic samples by interpolating between existing minority class instances instead of simply duplicating them. In our implementation, SMOTE (with random_state = 42) is applied to the encoded and scaled metadata features, guided by the diagnosis labels to identify minority classes. This process generates new synthetic feature samples for underrepresented diagnosis classes, effectively increasing both the number of metadata feature instances and the corresponding diagnosis labels. By balancing the dataset in this way, SMOTE enhances data diversity and improves the model’s ability to generalize across all classes.

In dermatological image analysis, datasets such as ISIC 2018 and ISIC 2019 frequently exhibit class imbalance, posing challenges for effective skin lesion classification. By applying oversampling techniques like SMOTE, the distribution of lesion types is balanced, leading to improved model accuracy, robustness, and generalization. Tables 3 and 4 present the class distribution before and after applying SMOTE to the ISIC 2018 and ISIC 2019 datasets, respectively, illustrating the impact of this technique in improving model training and prediction reliability.

**Table 3:** Class distribution before and after SMOTE on metadata of ISIC 2018 training dataset.

Class	Before oversampling	After oversampling
MEL	874	5,367
NV	5,367	5,367
BCC	408	5,367
AKIEC	265	5,367
BKL	893	5,367
DF	93	5,367
VASC	112	5,367
Total	8,012	37,569

**Table 4:** Class distribution before and after SMOTE on metadata of ISIC 2019 training dataset.

Class	Before oversampling	After oversampling
MEL	3,607	10,308
NV	10,308	10,308
BCC	2,665	10,308
AK	690	10,308
BKL	2,088	10,308
DF	191	10,308
VASC	191	10,308
SCC	524	10,308
Total	20,264	82,464

### Proposed model architecture

The proposed model integrates image data with structured metadata features to enhance skin lesion classification using an ensemble deep learning approach. It utilizes two publicly available datasets, ISIC 2018 and ISIC 2019, where ISIC 2019 contains additional metadata attributes such as age_approx, anatom_site_general, lesion_id, sex and malignancy status, which are absent in ISIC 2018. Given that ISIC 2018 is a subset of ISIC 2019, metadata was merged based on image names to create a unified dataset. The proposed model consists of three primary modules: a pre-processing module, a classification module, and a concatenation module.

The pre-processing module in the proposed model is divided into two components: image pre-processing and metadata pre-processing . In the image pre-processing stage, data augmentation techniques are applied to increase dataset diversity and improve model generalization. Additionally, all images are resized to 224 × 224 pixels to maintain consistent input dimensions across the deep learning models.

The metadata preprocessing process involves several key steps. First, only the most relevant metadata features are selected from the original dataset, including meta.clinical.age_approx, meta.clinical.anatom_site_general, meta.clinical.benign_malignant, meta.clinical.sex, and meta.clinical.diagnosis. Missing values in the numerical feature meta.clinical.age_approx are filled using the column mean, while missing values in categorical features such as meta.clinical.sex and meta.clinical.anatom_site_general are replaced with a default value of “unknown”. Categorical variables—including meta.clinical.sex, meta.clinical.anatom_site_general, and meta.clinical.benign_malignant—are transformed into numerical format using one-hot encoding. The numerical feature meta.clinical.age_approx is standardized using z-score normalization to ensure consistent scaling across inputs. The target labels for skin lesion diagnosis (meta.clinical.diagnosis) are also one-hot encoded to make them suitable for multi-class classification. Additionally, SMOTE (Synthetic Minority Over-sampling Technique) is applied to the training metadata and labels to address class imbalance by generating synthetic examples for underrepresented classes, improving the model’s ability to learn from all categories.

For classification, the proposed model employs a deep learning ensemble model consisting of ResNet50, Xception, and EfficientNetB0, where each model extracts deep image features through multiple layers. The GlobalAveragePooling2D layer reduces the spatial dimensions of the feature maps while preserving important information. A Dropout layer (0.5) is then applied to prevent overfitting by randomly deactivating neurons during training. Subsequently, a BatchNormalization layer stabilizes and accelerates the learning process by normalizing activations. Finally, a Dense layer with 64 neurons and ReLU activation captures high-level feature representations for the classification stage. The outputs from all models are fused using an adaptive weighted ensemble technique, where weights are dynamically learned based on each model’s validation performance, rather than being fixed or uniformly assigned.

This approach evaluates the reliability and predictive strength of each model during training and assigns higher weights to models that consistently perform better, while reducing the influence of weaker or less consistent ones. Unlike fixed or equal-weight averaging, the adaptive weighted ensemble enhances robustness by tailoring the contribution of each model to its actual effectiveness, leading to a more accurate and generalizable combined prediction. This dynamic weighting strategy strengthens the final image feature representation and improves the overall classification performance.

**Fig. 8:**
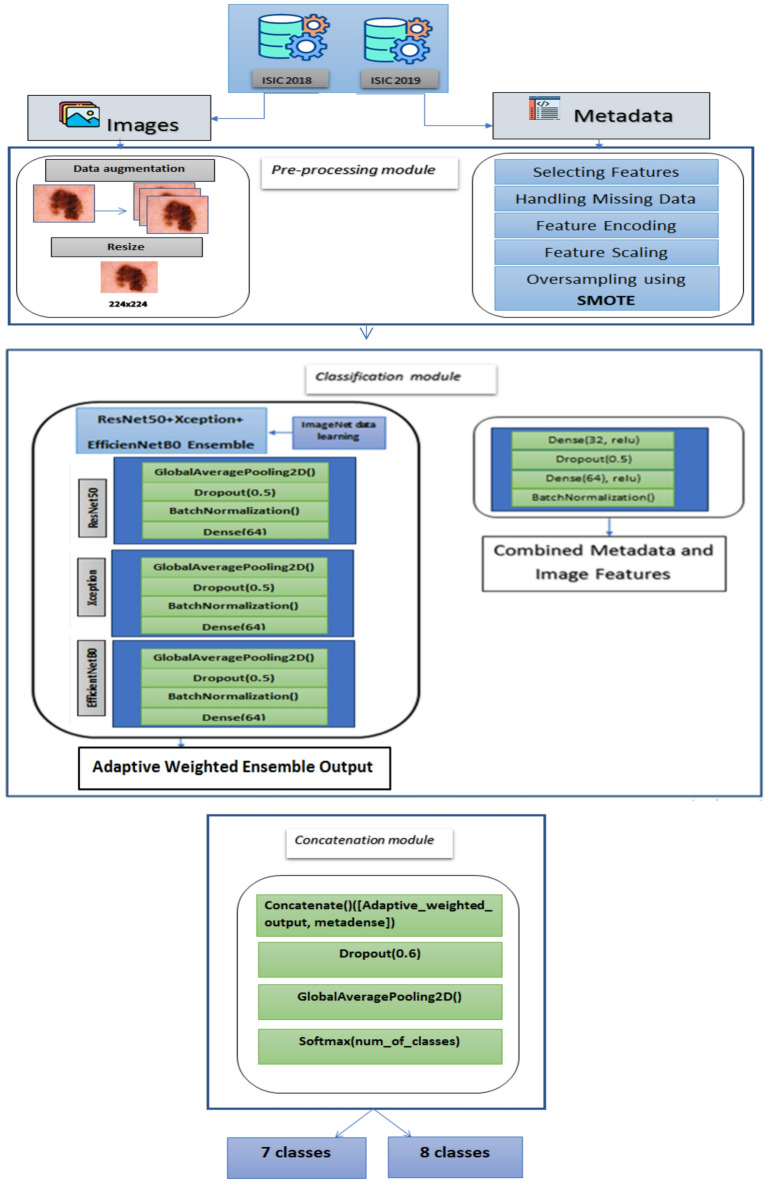
Workflow of the proposed model for classification for ISIC 2018 and ISIC 2019 Datasets.

Metadata features are processed through a dedicated neural network comprising Dense layers (32 and 64 neurons) with ReLU activation for non-linearity, Dropout (0.5) for regularization, and BatchNormalization to stabilize training. The fusion of image and metadata features is achieved through a concatenation layer, followed by Dropout (0.6) to reduce overfitting, GlobalAveragePooling2D for feature refinement, and a Softmax activation layer for final classification into 7 or 8 classes, depending on the dataset’s labelling scheme. The integration and fusion of image and metadata features significantly enhance classification accuracy compared to using image data alone, as metadata provides crucial contextual information that aids in distinguishing lesions with similar visual characteristics. The ensemble learning technique further improves robustness, demonstrating the effectiveness of combining deep learning-based image analysis with structured metadata to achieve superior diagnostic performance. Figure 8 shows the workflow of the proposed model classification process for ISIC 2018 and ISIC 2019 datasets.

## Experimental evaluation

This section provides an in-depth evaluation of the proposed model, including the experimental setup, evaluation metrics, benchmark models, experimental results and insights on the results. The methodology ensures a comprehensive assessment of the model’s performance and reliability. The details of this evaluation are presented in the following subsections.

### Experimental setup

A well-structured experimental setup is crucial for ensuring the reliability and effectiveness of the proposed model. This section outlines the training procedure setup and hardware and software setup, detailing the dataset split strategy, training configurations, and computational resources used for model evaluation.

#### Training procedure setup

The experimental setup involves a meticulous division the dataset into a training-validation-testing split ratio of 80:10:10, ensuring a comprehensive representation for model training and evaluation. Specifically, for the ISIC 2019 dataset, the split includes a training set of 20,264 images, a validation set of 2,533 images, and a testing set of 2,534 images. For the ISIC 2018 dataset, the split includes a total of 10,015 images before splitting, a training set of 8,012 images, a validation set of 1,001 images, and a testing set of 1,002 images. This approach allows the model to learn from a substantial portion of diverse data during training, while the remaining dataset portions serve as validation and testing sets to assess generalization and overall model performance.

We harness the computational power of Google Colab^45^ to efficiently execute the model training process, leveraging its cloud-based infrastructure. The applied parameters for training include steps per epoch set to 100, batch size of 32, total epochs of 100, a learning rate of 0.0001 (adjusted to improve convergence), and the Adam optimizer. To ensure optimal performance, early stopping is implemented with a patience of 10 epochs and a learning rate reduction strategy with a factor of 0.2 and patience of 5 epochs. All experiments were conducted using Google Colab with a Tesla T4 GPU, where the model—an ensemble of ResNet50 (~ 25.6 M parameters), Xception (~ 22.9 M), and EfficientNetB0 (~ 5.3 M)—was trained for 100 epochs This setup balances accuracy and efficiency, making it well-suited for high-memory GPUs and robust clinical image classification tasks. Table 5 shows the hyperparameters used in the experimental setup.

**Table 5:** Hyperparameters used in the experiments.

Parameter	Value
Steps per epoch	100
Batch size	32
Total epochs	100
Learning rate	0.0001
Optimizer	Adam
Early stopping patience	10 epochs
Learning rate reduction factor	0.2
LR reduction patience	5 epochs

#### Hardware and software setup

The experiments were conducted using a combination of cloud-based and local computing resources. Primarily, Google Colab was utilized, providing 12.7 GB of RAM. For enhanced computational capacity, Google Colab Pro was occasionally employed, offering 51 GB of RAM. Additionally, some experiments were performed on a local machine with the following specifications: Processor—Intel(R) Core(TM) i7-9700 CPU @ 3.00 GHz, Installed RAM—16.0 GB (15.8 GB usable), and a 64-bit operating system with an × 64-based processor. The software setup included TensorFlow 2.x, Keras, Python 3.x, and other necessary libraries. This setup leveraged both Google Colab’s cloud-based infrastructure and the local machine’s capabilities to ensure efficient model training and evaluation.

### Evaluation metrics

The evaluation of the proposed models involved a comprehensive set of metrics^46^ to gauge their performance in skin cancer detection. Accuracy, a fundamental measure, evaluates the total precision of the model’s predictions and is expressed as the proportion of successfully predicted occurrences to all instances.

1 $$accuracy =\frac{true \,positives + true\, negatives}{\begin{array}{c}total\, ins\mathrm{tan}ces\\ \end{array}}$$

Precision, a crucial metric, quantifies the model’s ability to correctly identify positive cases, minimizing false positives. It is computed as the ratio of true positive predictions to the sum of true positives and false positives.

2 $$prescision=\frac{true \,positives }{true\, positives + false\, positives}$$

Recall, or sensitivity, assesses how well the model can find all pertinent cases while reducing false negatives. The ratio of true positive predictions to the total of false negatives and true positives is used to calculate it.

3 $$recall =\frac{true\, positives}{true\, positives + false\, negatives}$$

The F1 score, a balanced metric combining (2) and (3), offers a holistic view of a model’s efficiency. It is calculated using precision and recall harmonic means.

4 $$F1 \,score = 2\cdot \frac{precision\cdot recall}{precision+recall}$$

Specificity measures the proportion of actual negative cases that are correctly identified by the model. It is especially important in medical diagnosis to avoid false alarms (false positives).

5 $$Specificity = \frac{true\, negatives}{true\, negatives+false\, positives}$$

AUC (Area Under the Curve) is a performance metric that measures a classifier’s ability to distinguish between classes. Specifically, it represents the probability that the model will rank a randomly chosen positive instance higher than a randomly chosen negative one.

These metrics collectively provide a robust assessment of the models’ effectiveness in skin cancer detection, offering insights into their accuracy, precision, recall, and overall performance.

### Benchmark approaches

A variety of benchmark deep learning models were employed in this study, selected for their architectural efficiency, innovative design, and established performance in image classification tasks. Many previous studies have utilized models pretrained on large-scale datasets and subsequently fine-tuned them for specific medical tasks. Pretrained models in medical disease diagnosis leverage transfer learning (TL)^47,48^, enabling the adaptation of knowledge acquired from extensive generic datasets to domain-specific medical applications. This approach significantly reduces computational requirements and training time, while enhancing model generalization on smaller, specialized datasets. However, without fine-tuning, models may not effectively capture disease-specific hierarchical feature patterns, resulting in reduced performance in medical diagnosis tasks^49^.

This diverse selection of models serves as a reference point for evaluating the effectiveness of skin cancer detection methods, providing valuable insights into the comparative strengths of different convolutional architectures. An ensemble strategy was also adopted to integrate the predictive capabilities of these models and enhance overall classification performance. The details of these models and the applied ensemble approach are discussed in the following subsections.

#### Xception

Xception^50^, short for "Extreme Inception," is a deep learning model introduced by Google. It is an extension of the Inception architecture, designed to improve the efficiency of learning representations. Xception employs depth-wise separable convolutions, which factorize the standard convolution into a depth-wise convolution and a pointwise convolution. This allows for more efficient use of parameters and reduces computational complexity. Xception has shown strong performance in image classification tasks and is known for its ability to capture intricate features in images.

#### ResNet50

ResNet50^51^ is a deep learning architecture that is part of the ResNet family, which introduced the concept of residual learning. Developed by Microsoft Research, ResNet50 is designed to solve the problem of training very deep neural networks by utilizing residual connections, also known as skip connections. These connections allow the network to learn residual functions, which makes it easier to train deeper models without facing the vanishing gradient problem. ResNet50, with its 50 layers, has been particularly successful in computer vision tasks such as image classification, object detection, and segmentation. By introducing batch normalization and other architectural innovations, ResNet50 achieves a balance between depth and performance, making it an efficient and widely adopted model for a range of applications in visual recognition.

#### MobileNet

MobileNet^52^ is a deep convolutional neural network design created especially for mobile and edge-based gadgets with constrained processing power to enable effective deployment. Introduced by Google researchers. MobileNet achieves a balance between accuracy and computational efficiency by utilizing depth-wise separable convolutions. This technique factorizes standard convolutions into depth-wise convolutions and pointwise convolutions, significantly reducing the number of parameters and computations. MobileNet is particularly well-suited for real-time image classification tasks on resource-constrained devices, making it a popular choice for mobile applications, embedded systems, and edge computing scenarios. Its lightweight nature and competitive performance have contributed to the widespread adoption of MobileNet as a go-to model for on-device machine learning applications.

#### EfficientNetB0

EfficientNetB0^53^ is the baseline model in the EfficientNet family, introduced by Google researchers. It is a convolutional neural network that achieves optimal performance by scaling the model’s depth, width, and resolution systematically using a compound scaling method. EfficientNetB0 achieves superior accuracy with fewer parameters and less computation compared to traditional architectures. By employing this compound scaling method, it manages to maintain a balance between efficiency and performance. Despite being the simplest model in the EfficientNet family, EfficientNetB0 has demonstrated excellent performance on image classification tasks while remaining computationally efficient, making it ideal for both resource-constrained environments and high-end applications. EfficientNetB0 has been widely adopted for various computer vision applications, including image classification and object detection.

#### DenseNet121

DenseNet121^54^ is a deep convolutional neural network model that is part of the DenseNet family. DenseNet121 addresses the vanishing gradient problem by employing dense connectivity, where each layer receives the feature maps from all preceding layers. This dense connectivity leads to improved gradient propagation, enhanced feature reuse, and efficient use of parameters. With 121 layers, DenseNet121 strikes a balance between depth and computational efficiency, providing superior performance on image classification tasks, object detection, and segmentation. The model’s densely connected architecture enables the creation of deeper and more efficient networks with fewer parameters compared to traditional architectures. As a result, DenseNet121 achieves competitive accuracy while being highly efficient, making it a strong candidate for modern computer vision applications.

#### Ensemble techniques

Ensemble techniques in convolutional neural networks (CNNs) aim to improve accuracy and generalization by combining predictions from multiple models. Common approaches include simple averaging and stacking, where a meta-learner combines base model outputs. Diversity among models—whether in architecture (e.g., ResNet, DenseNet), training setup, or data exposure—helps reduce individual model bias. These methods have proven effective in computer vision tasks and are widely used in challenges like ImageNet, where ensemble models often achieve top performance^55^.

More recently, adaptive weighted ensemble CNN models enhance prediction accuracy by combining multiple convolutional neural networks, each trained on the same task, and dynamically assigning weights to their outputs based on performance metrics such as confidence scores or validation accuracy. This adaptive mechanism allows the ensemble to emphasize models that perform better on specific inputs, often using attention-based strategies or meta-learners to optimize weight distribution. The final prediction is typically a weighted average of individual model outputs, resulting in improved generalization and robustness across diverse datasets. Such approaches have shown significant promise in applications like image classification, medical diagnosis, and deepfake detection, where model diversity and adaptability are crucial^56^.

### Experimental results

To comprehensively assess the performance of the proposed model, a rigorous set of experiments have been conducted on the ISIC 2018 and ISIC 2019 datasets as follows. Experiment 1 investigates the performance of five pre-trained state-of-the-art models— ResNet50, Xception, MobileNet, EfficientNetB0, and DenseNet121, and EfficientNetB0. This experiment focuses solely on image data, without incorporating metadata. In Experiment 2, transfer learning techniques were applied to incorporate metadata alongside image data for improved skin cancer classification. Experiment 3 aimed to further improve classification accuracy by employing an ensemble approach. Details on these experiments are presented in the following subsections.

#### Experiment 1

Experiment 1 investigates the performance of five pre-trained state-of-the-art models— ResNet50, Xception, MobileNet, EfficientNetB0, and DenseNet121, and EfficientNetB0—on the ISIC 2018 and ISIC 2019 datasets. This experiment focuses solely on image data, without incorporating metadata. Key evaluation metrics, including accuracy, precision, recall, and F1 score, are used to assess the models’ ability to classify skin cancer types. Tables 6 and 7 present the performance metrics before metadata integration for each model on the ISIC 2018 and ISIC 2019 datasets, respectively.

**Table 6:** The experimental results on ISIC 2018 using pre-trained models before metadata integration.

Model Name	Accuracy	Precision	Recall	F1 score
MobileNet	0.776	0.82	0.77	0.78
Xception	0.834	0.85	0.83	0.84
ResNet50	0.825	0.85	0.82	0.83
EfficientNetB0	0.823	0.82	0.82	0.81
DenseNet121	0.814	0.85	0.81	0.82

**Table 7:** The experimental results on ISIC 2019 using pre-trained models before metadata integration.

Model Name	Accuracy	Precision	Recall	F1 score
MobileNet	0.732	0.72	0.72	0.71
Xception	0.834	0.83	0.83	0.83
ResNet50	0.787	0.78	0.79	0.78
EfficientNetB0	0.821	0.80	0.82	0.82
DenseNet121	0.772	0.76	0.77	0.76

For ISIC 2018, Xception achieved the highest performance with an accuracy of 83.4% and an F1 score of 84%, followed closely by ResNet50 and EfficientNetB0, which also maintained balanced precision and recall. In contrast, MobileNet recorded the lowest performance, with an accuracy of 77.6% and an F1 score of 78%. For the ISIC 2019, Xception again led with both accuracy and F1 score at 83.4%, indicating strong generalization across datasets. EfficientNetB0 also performed well (accuracy: 82.1%, F1 score: 82%), whereas MobileNet had the weakest performance, with an accuracy of 73.2% and an F1 score of 71%.

Overall, the results show that Xception consistently outperformed the other models across both datasets, particularly in terms of F1 score. On the other hand, MobileNet showed limitations, likely due to its lightweight architecture, which may sacrifice representational depth. These baseline metrics serve as a clear reference point to assess the improvements introduced by metadata integration in the following experiment.

#### Experiment 2

Experiment 2 presents a metadata-aware ablation study, it focuses on evaluating the impact of metadata integration on the performance of five pre-trained models—ResNet50, Xception, MobileNet, EfficientNetB0, and DenseNet121—using the ISIC 2018 and ISIC 2019 datasets. Transfer learning techniques were applied to incorporate metadata alongside image data for improved skin cancer classification. Pre-processing steps such as oversampling with SMOTE and handling missing values were employed to address class imbalance and enhance the models’ generalization capabilities. Tables 8 and 9 present the performance metrics after metadata integration for the ISIC 2018 and ISIC 2019 datasets, respectively. These results highlight the significant improvement in model performance achieved through the integration of metadata and robust pre-processing . This experiment clearly demonstrates the value of combining metadata with image data, which contributes to more accurate and reliable skin cancer classification.

**Table 8:** The experimental results on ISIC 2018 using pre-trained models after metadata integration.

Model Name	Accuracy	Precision	Recall	F1 score
MobileNet	0.816	0.86	0.81	0.82
Xception	0.880	0.87	0.88	0.88
ResNet50	0.875	0.88	0.87	0.85
EfficientNetB0	0.897	0.88	0.89	0.89
DenseNet121	0.854	0.86	0.85	0.85

**Table 9:** The experimental results on ISIC 2019 using pre-trained models after metadata integration.

Model name	Accuracy	Precision	Recall	F1 score
MobileNet	0.764	0.76	0.76	0.75
Xception	0.863	0.85	0.86	0.86
ResNet50	0.832	0.82	0.83	0.83
EfficientNetB0	0.878	0.88	0.87	0.87
DenseNet121	0.794	0.78	0.79	0.78

After metadata integration, a noticeable improvement in performance was observed across all models for both ISIC 2018 and ISIC 2019 datasets. On the ISIC 2018 dataset, EfficientNetB0 achieved the highest overall performance, with an accuracy of 89.7%, recall of 89%, and an F1 score of 89%, indicating a strong balance between precision and recall. Xception and ResNet50 also showed significant gains, with accuracies of 88.0% and 87.5%, respectively. Even MobileNet, which previously underperformed, improved its accuracy from 77.6% to 81.6%, reflecting the positive impact of metadata inclusion.

Similarly, for ISIC 2019, EfficientNetB0 continued to lead, reaching an accuracy of 87.8% and an F1 score of 87%. Xception also maintained strong performance (accuracy: 86.3%, F1 score: 86%), while ResNet50 improved to an accuracy of 83.2%. Notably, MobileNet showed a modest but meaningful increase in performance, improving from 73.2% to 76.4% accuracy. These results clearly demonstrate that metadata integration significantly enhanced model performance, especially for models like MobileNet and DenseNet121 that had relatively lower baseline scores. The consistent improvement across all architectures reinforces the importance of incorporating patient metadata in medical image classification tasks for more accurate and reliable results.

Furthermore, paired t-tests were conducted to evaluate whether integrating metadata significantly improved the performance of five pretrained models on the ISIC 2018 and ISIC 2019 datasets. The performance metrics before and after metadata fusion for each model were treated as paired samples to directly assess these changes. Tables 10 and 11 below summarize the t-statistics, *p*-values, and the statistical significance for key metrics including Accuracy, Precision, Recall, and F1 Score of ISIC 2018 and ISIC 2019 datasets.

**Table 10:** Paired T-test results showing performance improvements after metadata fusion on ISIC 2018 dataset.

Metric	t-statistic	*p*-value	Statistical Significance
Accuracy	7.9455	0.0014	Statistically significant (*p* < 0.05)
Precision	3.7199	0.0205	(*p* < 0.05)
Recall	9.1287	0.0008	(*p* < 0.05)
F1 Score	4.1184	0.0146	(*p* < 0.05)

**Table 11:** Paired T-test results showing performance improvements after metadata fusion on ISIC 2019 dataset.

Metric	t-statistic	*p*-value	Statistical Significance
Accuracy	5.9324	0.0040	Statistically significant (*p* < 0.05)
Precision	3.6515	0.0217	(*p* < 0.05)
Recall	7.0602	0.0021	(*p* < 0.05)
F1 Score	6.5169	0.0029	(*p* < 0.05)

The paired t-test results demonstrate statistically significant improvements across all four metrics after metadata fusion. In both datasets, all four evaluated metrics—Accuracy, Precision, Recall, and F1 Score—show statistically significant improvements with *p*-values well below the 0.05 threshold. Notably, Recall demonstrates the highest t-statistic in both datasets (9.1287 for ISIC 2018 and 7.0602 for ISIC 2019), indicating that metadata fusion most strongly enhances the model’s ability to correctly identify positive cases. Accuracy follows closely, with t-values of 7.9455 (ISIC 2018) and 5.9324 (ISIC 2019), reflecting consistent improvements in overall classification correctness. Precision and F1 Score also show meaningful gains in both datasets, confirming that metadata contributes to reducing false positives while maintaining a balanced performance.

The statistical analysis across both datasets provides robust evidence that integrating metadata leads to significant and consistent enhancements in model performance, particularly in recall and accuracy. This supports the value of metadata fusion as an effective strategy for improving deep learning models in dermatological image analysis.

#### Experiment 3

Following the integration of metadata in Experiment 2, which significantly enhanced the performance of individual models, Experiment 3 aimed to further improve classification accuracy by employing an ensemble approach. The top-performing models from the previous experiment—ResNet50, Xception, and EfficientNetB0—were selected based on their strong individual performance. We explored three ensemble methods: unweighted (simple) averaging, stacking, and an adaptive weighted ensemble technique. The advanced weighted averaging method dynamically assigns weights to each model’s predictions based on their individual performance and reliability, rather than using fixed or equal weights. This adaptive weighting allows the ensemble to better leverage the strengths of each model while minimizing the impact of weaker predictions, resulting in improved robustness and accuracy. These ensemble techniques were combined to capitalize on complementary features from different architectures, and this strategy proved highly effective, achieving superior results across both datasets.

**Fig. 9:**
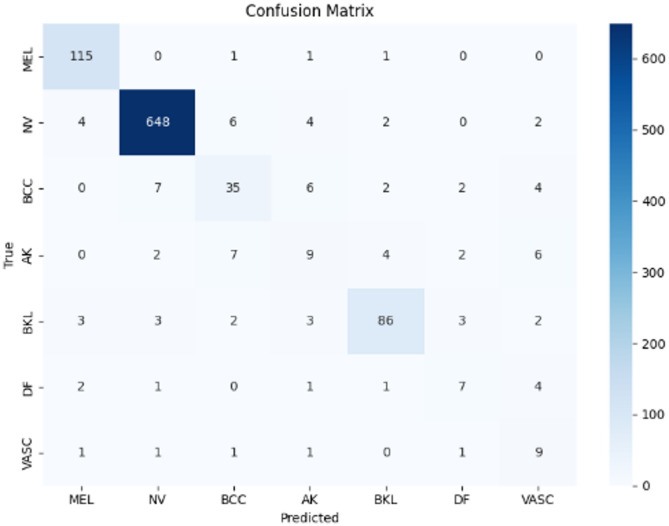
Confusion matrix using stacking technique on ISIC 2018 dataset.

**Fig. 10:**
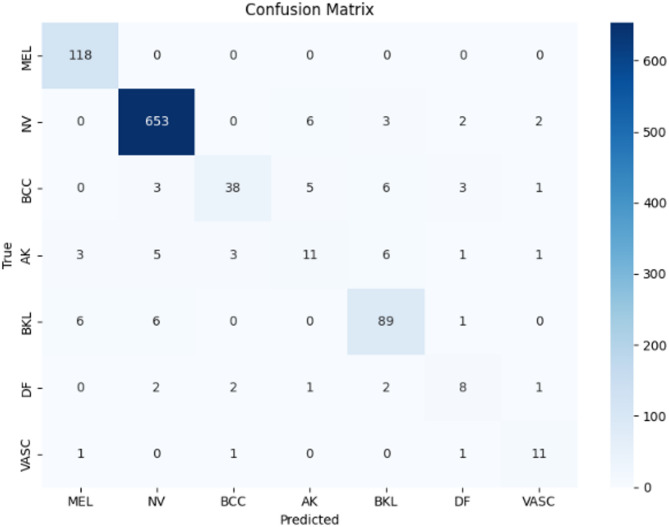
Confusion matrix of the simple average on ISIC 2018 dataset.

**Fig. 11:**
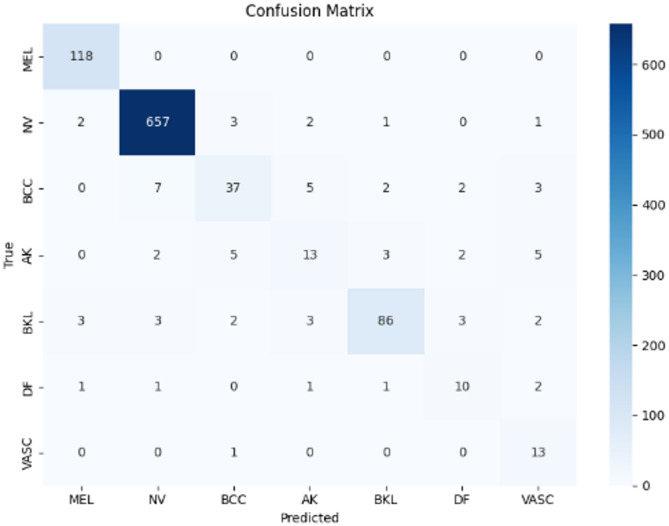
Confusion matrix using proposed adaptive weighted ensemble on ISIC 2018 dataset.

**Fig. 12:**
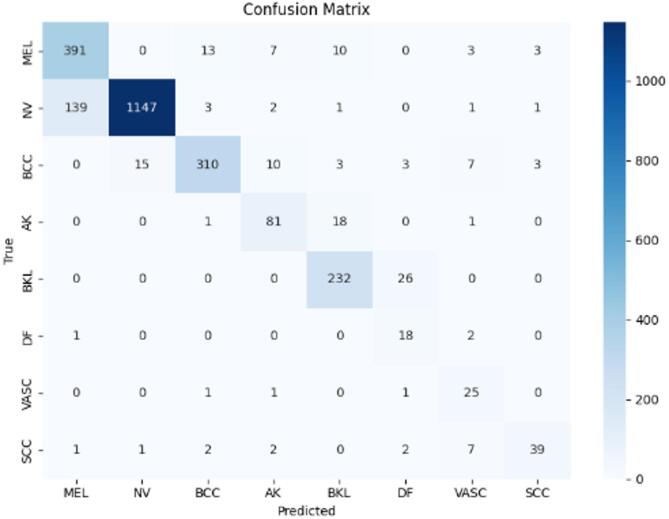
Confusion matrix using stacking technique on ISIC 2019 dataset.

**Fig. 13:**
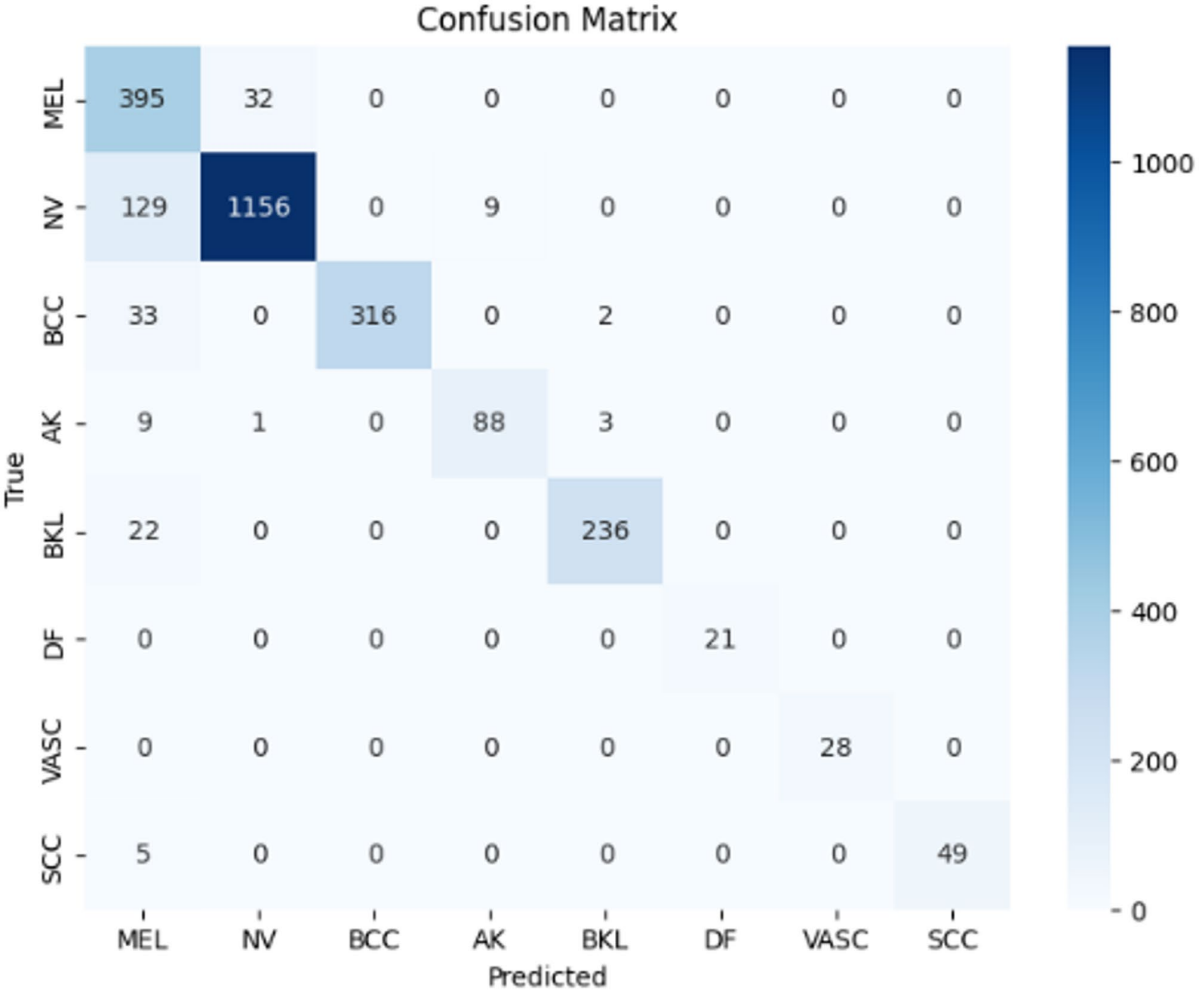
Confusion matrix of the simple average on ISIC 2019 dataset.

**Fig. 14:**
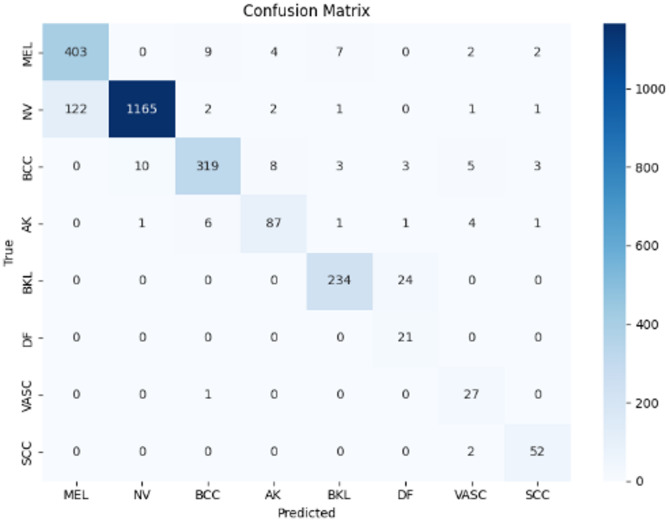
Confusion matrix using proposed adaptive weighted ensemble on ISIC 2019 dataset.

Figures 9, 10, and 11 illustrate the confusion matrices generated using the stacking, unweighted simple average, and adaptive weighted ensemble methods, respectively, on the ISIC 2018 dataset. Likewise, Figs. 12, 13, and 14 present the corresponding confusion matrices for the ISIC 2019 dataset. These visualizations highlight the robustness of the ensemble approaches in addressing the challenges of multi-class skin lesion classification involving 7 and 8 classes.

Tables 12 and 13 summarize the performance of three ensemble techniques—simple average, stacking, and adaptive weighted ensemble for the ISIC 2018 and ISIC 2019 datasets, respectively. Table 12 presents the evaluation metrics for different ensemble techniques applied to the ISIC 2018 dataset. The proposed adaptive weighted ensemble achieved the highest overall performance, with an accuracy of 93.2%. It showed a slight improvement of approximately 0.6% over the simple average ensemble (92.6%) and a more notable improvement of about 2.5% compared to the stacking method (90.7%). The proposed ensemble using the adaptive weighted technique and the simple average ensemble produced relatively close results, particularly in precision, recall, and F1 score. However, the proposed technique maintained a consistent edge, achieving 93% precision and 93% F1 score. Although the loss value was slightly higher (0.225 vs. 0.205 in the simple average), the proposed method achieved the highest AUC of 97.3%, indicating stronger overall classification confidence. Overall, while both simple and adaptive weighted methods performed similarly, the proposed adaptive weighted ensemble demonstrated the most reliable and superior results across all metrics.

**Table 12:** Evaluation metrics for the ensemble techniques on ISIC 2018 dataset.

Ensemble technique	Accuracy	Precision	Recall	F1 score	Loss	AUC
Stacking	0.907	0.91	0.91	0.91	0.357	0.940
Simple Average	0.926	0.92	0.93	0.92	0.205	0.962
**Proposed adaptive weighted ensemble**	**0.932**	**0.93**	**0.93**	**0.93**	**0.225**	**0.973**

**Table 13:** Evaluation metrics for the ensemble techniques on ISIC 2019 dataset.

Ensemble technique	Accuracy	Precision	Recall	F1 score	Loss	AUC
Stacking	0.885	0.90	0.89	0.89	0.405	0.905
Simple average	0.903	0.92	0.90	0.91	0.319	0.948
**Proposed adaptive weighted ensemble**	**0.911**	**0.93**	**0.91**	**0.92**	**0.306**	**0.955**

Similar to the trends observed on the ISIC 2018 dataset, Table 13 shows that the stacking ensemble again delivered the weakest performance on ISIC 2019, with an accuracy of 88.5%, precision of 90%, and an F1 score of 89%. In contrast, the simple average ensemble improved these results, pushing accuracy to 90.3% and F1 score to 91%, while also reducing the loss from 0.405 to 0.319. The proposed adaptive weighted ensemble achieved the strongest results overall, reaching an accuracy of 91.1%, precision of 93%, and F1 score of 92%. It also achieved the lowest loss (0.306) and the highest AUC (95.5%), indicating more confident and consistent predictions. These results reinforce the effectiveness of the proposed method, consistently outperforming traditional ensemble strategies across different datasets.

Tables 14 and 15 show the evaluation metrics of the proposed ensemble model per each class in ISIC 2018 and ISIC 2019 datasets respectively.

**Table 14:** Evaluation metrics for the proposed ensemble per each class of ISIC 2018 dataset.

Class	TP	FP	FN	TN	Precision	Recall	F1 Score	Specificity	AUC
MEL	118	6	0	882	0.952	1.000	0.975	0.993	0.997
NV	657	14	9	326	0.981	0.986	0.984	0.961	0.973
BCC	37	14	19	936	0.771	0.661	0.712	0.988	0.968
AK	13	11	19	983	0.542	0.433	0.481	0.989	0.964
BKL	86	7	16	918	0.925	0.843	0.882	0.992	0.971
DF	10	7	6	1007	0.588	0.625	0.606	0.993	0.969
VASC	13	13	1	1007	0.500	0.929	0.650	0.987	0.971

**Table 15:** Evaluation metrics for the proposed ensemble per each class of ISIC 2019 dataset.

Class	TP	FP	FN	TN	Precision	Recall	F1 Score	Specificity	AUC
MEL	403	122	26	2545	0.768	0.944	0.847	0.942	0.953
NV	1165	11	129	1791	0.991	0.900	0.943	0.991	0.974
BCC	319	18	32	2727	0.947	0.909	0.927	0.992	0.936
AK	87	14	14	2981	0.861	0.861	0.861	0.994	0.923
BKL	234	12	24	2826	0.951	0.907	0.929	0.995	0.954
DF	21	3	0	3072	0.429	1.000	0.600	0.989	0.951
VASC	27	14	9	3046	0.659	0.964	0.783	0.994	0.984
SCC	52	7	2	3035	0.881	0.963	0.920	0.997	0.969

The proposed ensemble model demonstrated high classification performance on the ISIC 2018 dataset as shown in Table 14, particularly for prevalent and high-risk skin lesion types. For Melanoma (MEL), the model achieved a perfect recall of 1.000, indicating that all actual MEL cases were correctly identified, alongside a high specificity of 0.993, which reflects minimal misclassification of non-MEL cases. Nevus (NV) classification was also highly accurate with a recall of 0.986 and a precision of 0.981, resulting in an F1 score of 0.984. Basal Cell Carcinoma (BCC) showed more modest results, with a recall of 0.661 and a specificity of 0.988, suggesting that while the model accurately excluded non-BCC cases, it missed a considerable number of true positives. Actinic Keratoses (AK) and Dermatofibroma (DF) had the lowest F1 scores (0.481 and 0.606, respectively), reflecting difficulty in recognizing these underrepresented classes. Despite this, the model maintained high specificity (above 0.98) across all classes, meaning it was effective at ruling out incorrect classifications even when sensitivity varied.

Table 15 presents the model’s performance on the ISIC 2019 dataset, which contains more classes and greater complexity. The model continued to perform strongly for major categories. MEL achieved a high recall of 0.944, although with a reduced precision of 0.768 due to an increase in false positives. NV maintained excellent precision (0.991) but experienced a lower recall (0.900), leading to missed true cases. Performance on BCC and BKL was consistent and reliable, with F1 scores above 0.92 and specificity near-perfect at over 0.99. Notably, AK saw significant improvement compared to ISIC 2018, achieving balanced precision and recall (both at 0.861). For rare classes, the model showed mixed outcomes: DF achieved a perfect recall of 1.000, meaning no actual DF cases were missed, but low precision (0.429) due to false positives. VASC also showed strong recall (0.964) but moderate precision (0.659). The newly included class, Squamous Cell Carcinoma (SCC), was well classified with an F1 score of 0.920 and specificity of 0.997, indicating the model’s ability to adapt to new lesion types.

**Fig. 15:**
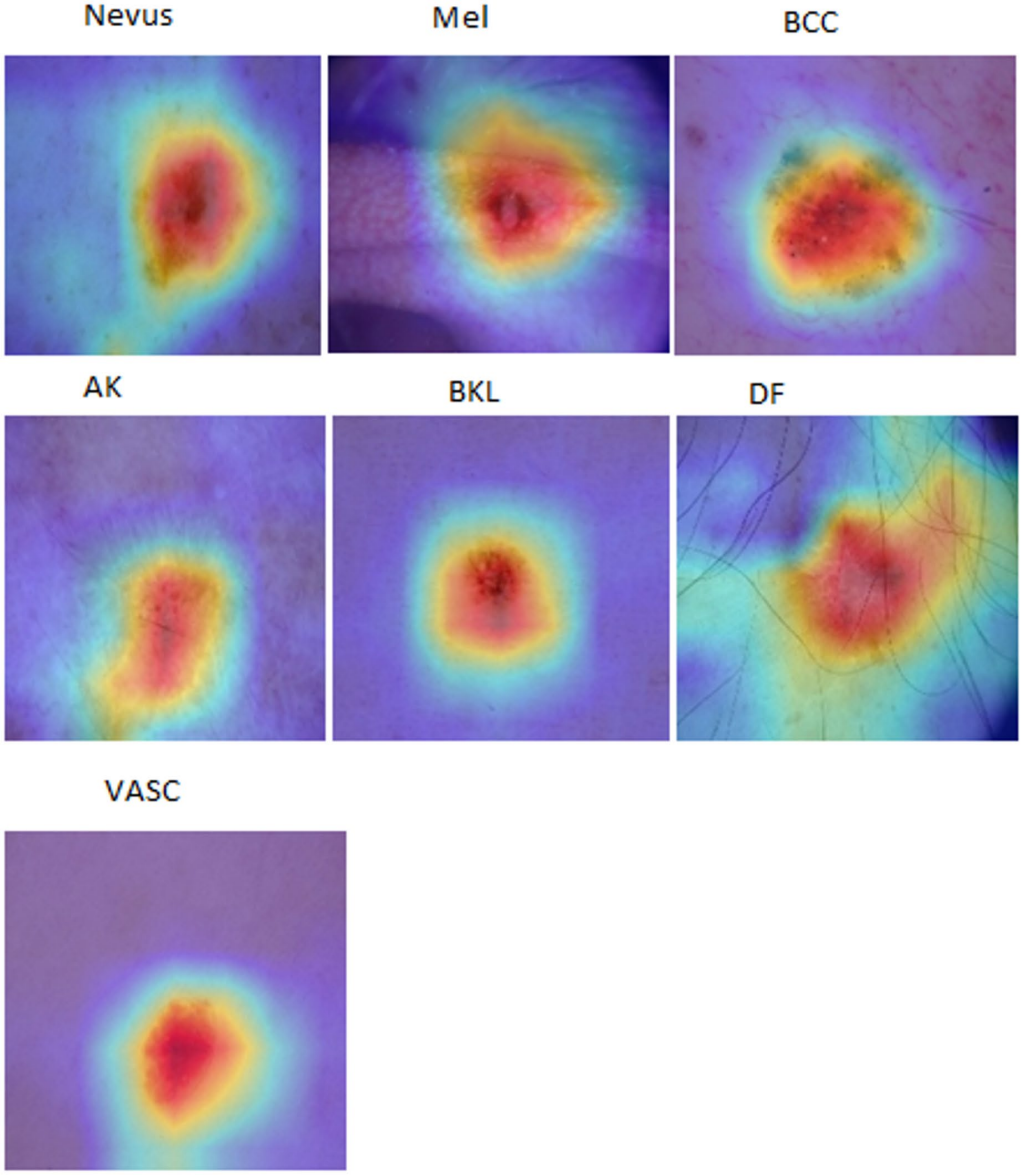
Grad-CAM visualizations of ISIC 2018 dataset.

**Fig. 16:**
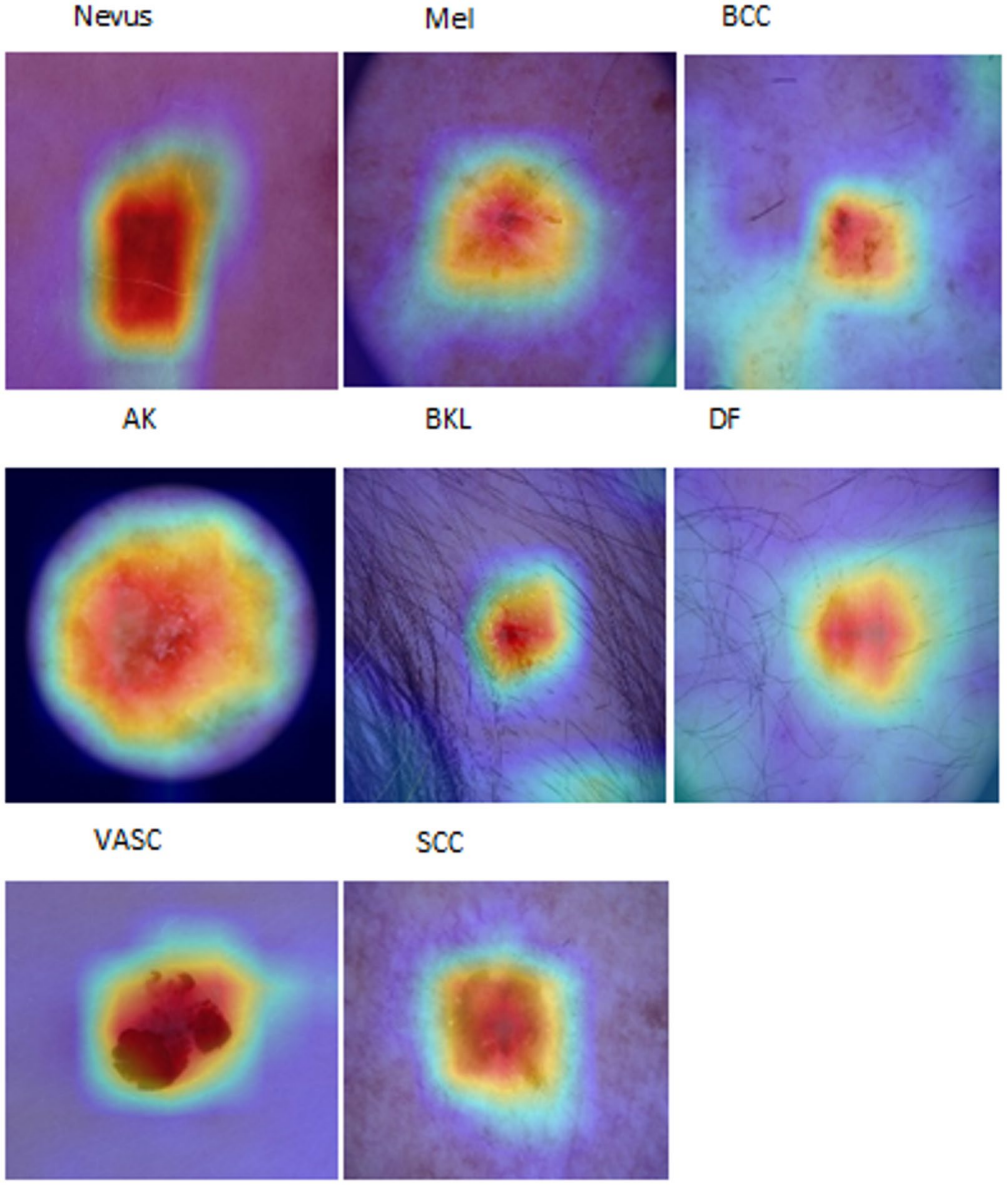
Grad-CAM visualizations of the ISIC 2019 dataset.

Furthermore, Figs. 15 and 16 showcase Grad-CAM visualizations for representative samples from the ISIC 2018 and ISIC 2019 datasets, respectively. These visual explanations are critical for understanding the model’s decision-making process, as they highlight the most salient regions in the input images that contribute to the final classification. The inclusion of Grad-CAM enhances model interpretability and supports clinical trust by aligning model attention with relevant dermatological features. Overall, the combined analysis of performance metrics and visual explanations underscores the impact of the proposed ensemble framework—particularly when integrated with metadata—in improving both the accuracy and transparency of automated skin lesion classification.

**Fig. 17:**
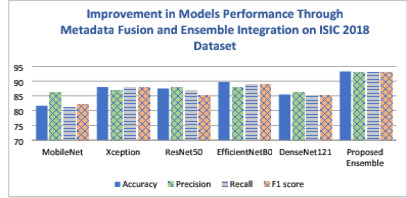
Models performance after metadata fusion and ensemble integration on ISIC 2018 dataset.

**Fig. 18:**
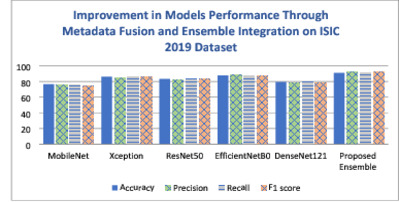
Models performance after metadata fusion and ensemble integration on ISIC 2019 dataset.

Additionally, Fig. 17 shows the performance of the individual models alongside the proposed ensemble after applying metadata integration on the ISIC 2018 dataset, while Fig. 18 presents the corresponding results on the ISIC 2019 dataset, emphasizing the contribution of the ensemble technique in enhancing classification performance across both datasets.

Figures 17 and 18 illustrate the progressive improvement in model performance following the integration of metadata features and the development of the proposed adaptive weighted ensemble model. The bar charts clearly demonstrate a consistent enhancement across all models after metadata fusion, confirming that combining clinical metadata with image features enriches the learning process and improves classification accuracy. In Fig. 17, corresponding to the ISIC 2018 dataset, each baseline model exhibits a noticeable accuracy increase after metadata integration, with the adaptive weighted ensemble achieving the highest overall performance. Similarly, Fig. 18 presents the results for the ISIC 2019 dataset, where a comparable upward trend is observed, emphasizing the robustness and generalizability of the fusion strategy across different datasets. The adaptive weighted ensemble, which dynamically assigns optimal weights to each base learner, further refines prediction confidence and yields superior accuracy compared to individual models and simple averaging ensembles. Overall, these results highlight the effectiveness of metadata fusion and adaptive weighting in significantly enhancing the discriminative capability of deep learning models for skin cancer classification.

#### Proposed model validation on Derm7pt dataset

To further validate the robustness and generalizability of our proposed ensemble, we evaluated its performance on an independent external dataset, Derm7pt^43^. Derm7ptis a publicly available dermoscopic image dataset commonly used for skin lesion classification. We identified six common classes between Derm7pt and the combined ISIC 2018 & 2019 datasets, enabling a fair comparative evaluation. These classes include all ISIC 2018 classes except Actinic Keratosis (AKIEC). The six common classes are: Melanocytic Nevi (NV), Melanoma (MEL), Basal Cell Carcinoma (BCC), Benign Keratosis-like Lesions (BKL), Dermatofibroma (DF), and Vascular Lesions (VASC).

Our proposed ensemble was trained on six common lesion classes from the ISIC 2018 and 2019 datasets, achieving a high training accuracy of 98.3% and a validation accuracy of 95.4%. It was then tested on the Derm7pt dataset without any additional training or fine-tuning, ensuring an unbiased assessment of its performance on unseen data. The results demonstrate that the metadata fusion approach incorporated in our ensemble significantly enhances its ability to generalize beyond the original ISIC datasets, maintaining competitive accuracy and robust performance across all evaluation metrics. Specifically, the ensemble achieved an accuracy of 82.5%, with a precision of 86%, recall of 83%, F1 score of 84%, and AUC of 89.15%, indicating a strong balance between sensitivity and specificity.

**Fig. 19:**
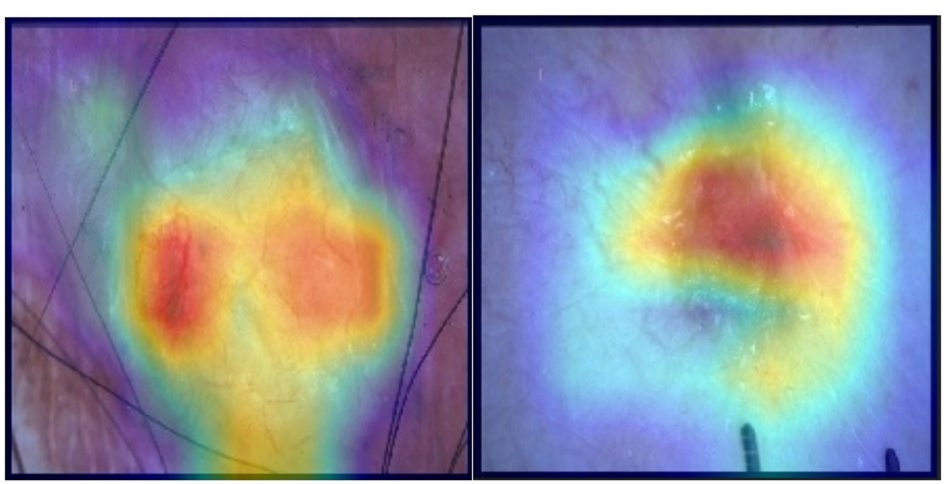
Grad-CAM visualization sample of Derm7pt dataset.

**Fig. 20:**
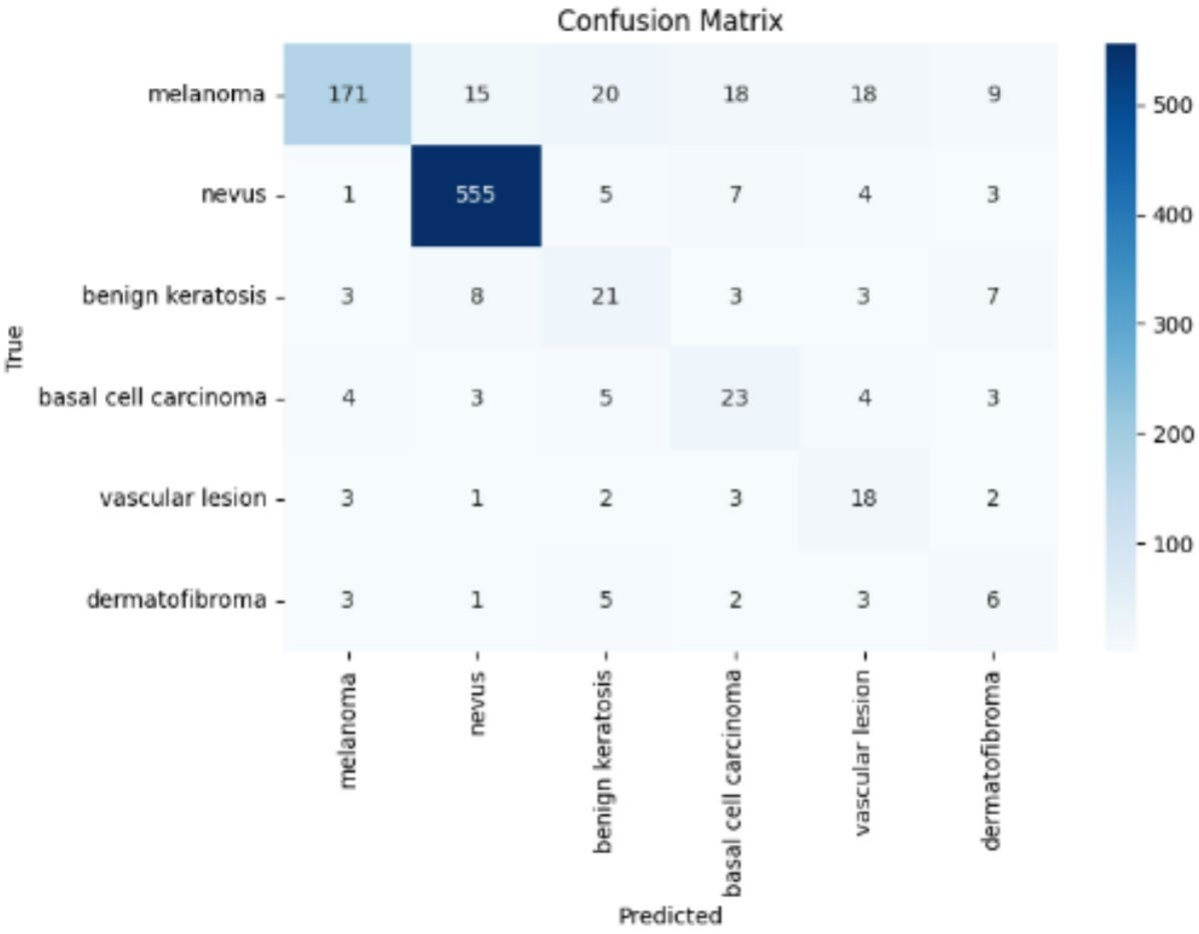
Confusion matrix of Derm7pt dataset after using the proposed ensemble model.

The observed drop in accuracy from 95.4% (validation on ISIC) to 82.5% (external Derm7pt) reflects the natural challenge of applying models trained on one dataset to a different, unseen dataset with possible variations in image quality, acquisition conditions, and class distributions. Despite this decline, the model maintains strong precision and recall, suggesting it still effectively identifies skin lesion types with relatively few false positives and false negatives. This highlights the robustness and generalizability of the ensemble and metadata fusion approach, which helps mitigate performance degradation when encountering diverse real-world data. Table 16 summarizes the per-class performance, including precision, recall, F1 score, and specificity for each lesion category. Figure 19 shows the Grad-CAM Visualization Sample of Derm7pt Dataset while Fig. 20 illustrates the confusion matrix, providing a visual representation of the model’s classification behaviour across the six classes.

**Table 16:** Evaluation metrics of each class in Derm7pt dataset using the proposed model.

Class	TP	FP	FN	TN	Precision	Recall	F1 Score	Specificity	AUC
Melanoma	171	14	80	697	0.9243	0.6813	0.7844	0.9803	0.9235
Nevus	555	28	20	359	0.9520	0.9652	0.9585	0.9276	0.8450
Benign Keratosis	21	37	24	880	0.3621	0.4667	0.4078	0.9597	0.9025
Basal Cell Carcinoma	23	33	19	887	0.4107	0.5476	0.4694	0.9641	0.8515
Vascular Lesion	18	32	11	901	0.3600	0.6207	0.4557	0.9657	0.9925
Dermatofibroma	6	24	14	918	0.2000	0.3000	0.2400	0.9745	0.8340

### Insights on experimental results

In Experiments 1 and 2, five pre-trained models— ResNet50, Xception, MobileNet, EfficientNetB0, and DenseNet121—were employed, utilizing transfer learning to create a robust model for skin cancer detection. A key feature of this approach was the integration of metadata with image data, evaluated on two datasets: ISIC 2018 and ISIC 2019. This integration led to significant improvements in model accuracy, precision, recall, and F1 score.

The metadata, encompassing patient demographics and lesion characteristics, was seamlessly fused with the high-dimensional image features extracted by the pre-trained models. This fusion involved concatenating metadata vectors with deep features learned by the neural networks, allowing the models to leverage both visual and contextual information. To address class imbalance in the metadata, SMOTE was applied to oversample underrepresented categories, and missing data was handled using imputation methods to ensure no crucial information was lost. Data augmentation techniques were applied exclusively to the image data, enhancing the diversity of the training set and improving the models’ generalization.

Together, these strategies—transfer learning, metadata integration, SMOTE, data augmentation, and regularization—improved the model performance by preventing overfitting, enhancing generalization, and providing more reliable predictions for skin cancer detection.

To further improve the accuracy, an ensemble technique was employed in Experiment 3, combining the top-performing models— ResNet50, Xception, and EfficientNetB0. This ensemble approach mitigated individual model biases, reduced prediction variance, and increased diagnostic reliability. With metadata integration, the ensemble model achieved an impressive final accuracy of 93.2% on the ISIC 2018 dataset and 91.1% on the ISIC 2019 dataset, demonstrating the efficacy of combining model ensemble, metadata utilization, and advanced data handling techniques for enhanced performance.

On the ISIC 2018 dataset, the integration of metadata led to notable performance improvements across all models. MobileNet achieved a 5.15% increase in accuracy, with gains of 4.88% in precision, 5.19% in recall, and 5.13% in F1 score. Xception improved by 5.52% in accuracy, 2.35% in precision, 6.02% in recall, and 4.76% in F1 score. ResNet50 showed a 6.06% accuracy increase, along with improvements of 3.53% in precision, 6.1% in recall, and 2.41% in F1 score. EfficientNetB0 experienced the most significant boost, with a 9.00% increase in accuracy, 7.32% in precision, 8.54% in recall, and 9.88% in F1 score. DenseNet121 also showed solid performance gains, improving by 4.91% in accuracy, 1.18% in precision, 4.94% in recall, and 3.66% in F1 score. These consistent enhancements—particularly in recall and F1 score—underscore the effectiveness of metadata integration in refining deep learning model performance for medical image classification.

Furthermore, the ensemble model combining ResNet50, Xception, and EfficientNetB0, which were the top-performing individual models, benefited substantially from metadata integration, achieving a final accuracy of 93.2% and AUC of 97.3%. This result highlights how the fusion of metadata and ensemble learning significantly enhances generalization and robustness in classifying skin lesions, contributing to more accurate and reliable outcomes in medical image analysis.

Similarly, on the ISIC 2019 dataset, the integration of metadata proved particularly beneficial, emphasizing the importance of additional contextual information to enhance each model’s predictive capabilities. Accuracy improvements after metadata integration included 4.37% for MobileNet, with 5.56% precision, 5.56% recall, and 5.63% F1 score; 3.47% for Xception, with 2.41% precision, 3.61% recall, and 3.61% F1 score; 5.72% for ResNet50, with 5.13% precision, 5.06% recall, and 6.41% F1 score; 6.94% for EfficientNetB0, with 10.00% precision, 6.1% recall, and 6.1% F1 score; and 2.85% for DenseNet121, with 2.63% precision, 2.6% recall, and 2.63% F1 score. These improvements demonstrate the impact of metadata and pre-processing techniques in elevating model performance across critical evaluation metrics. The highest accuracy was achieved through an ensemble of ResNet50, Xception, and EfficientNetB0, combined using an adaptive weighted ensemble technique. This ensemble method achieved 91.1% accuracy and AUC of 95.5%, outperforming all individual models. The effectiveness of this approach highlights the strength of fusing multiple high-performing models, offering a balanced and computationally efficient strategy for improving classification performance in skin cancer detection. The results emphasize the effectiveness of ensemble learning in boosting performance and advancing medical image analysis.

The experimental results in Experiment 3 clearly demonstrate the superiority of the proposed adaptive weighted ensemble over both stacking and simple averaging methods on the ISIC 2018 and ISIC 2019 datasets. On ISIC 2018, the proposed method achieved an accuracy of 93.2%, representing a 2.76% improvement over stacking and a 0.65% gain over simple averaging. Similarly, the AUC improved by 3.51% compared to stacking and 1.14% over simple averaging, confirming enhanced discriminative power. Other metrics such as precision, recall, and F1 score all reached 93%, showing consistent performance gains. On ISIC 2019, the model again outperformed its counterparts, achieving 91.1% accuracy, which is 2.94% higher than stacking and 0.88% higher than simple averaging. The AUC on this dataset improved by 5.52% over stacking and 0.74% over simple averaging. These consistent improvements across all evaluation metrics indicate that the adaptive weighting mechanism effectively balances the strengths of individual models, leading to better ensemble behaviour, reduced misclassification, and improved generalization. The results highlight not only the robustness of the proposed method but also its practical relevance in real-world dermatological diagnosis tasks.

The proposed ensemble model was trained on six common lesion classes from the ISIC 2018 and 2019 datasets. It achieved a high training accuracy of 98.3% and a validation accuracy of 95.4%, with training accuracy even higher, indicating strong performance on the source data. To assess its generalization capability, the model was evaluated directly on the external Derm7pt dataset without any additional training or fine-tuning, ensuring a fully unbiased test. Despite natural domain differences, the model maintained robust performance on unseen data, achieving 82.5% accuracy, 86% precision, 83% recall, 84% F1 score, and an AUC of 0.8915. This ~ 13% drop from validation to external accuracy is within acceptable limits for cross-dataset evaluation in medical imaging. The inclusion of patient metadata significantly enhanced the model’s ability to differentiate between lesion types with similar visual characteristics. These results confirm the model’s generalizability, supported by per-class metrics, Grad-CAM visualizations, and a confusion matrix that further illustrate its classification behaviour and interpretability.

### Comparative analysis

To evaluate the proposed model ‘s performance, a comparative analysis against relevant studies conducted on ISIC 2018 and ISIC 2019 datasets is performed. For the ISIC 2018 competition, balanced accuracy was used as the primary evaluation metric. To contextualize our findings, Table 17 provides a comparative summary of related studies and their results on ISIC 2018 and ISIC 2019, highlighting the advancements in the field and positioning our approach within the broader research landscape.

**Table 17:** Comparative analysis against relevant studies on ISIC 2018 and ISIC 2019 datasets.

Study	Dataset	Pre-processing	Use metadata	Classifier and training algorithm	Parameters	Accuracy (%)	Proposed model
^30^	ISIC 2018	Data augmentation, colour constancy (Shades of Gray), metadata encoding	Yes	EfficientNet-B3 & B4 with metadata fusion, TTA	SGD, OneCycle LR, weighted cross-entropy loss	89.5%	**93.2%**
^31^		Image: Data augmentation Metadata: Handling missing values, normalization of numerical attributes (age), balancing metadata distribution	Yes	Hybrid CNN-ViT with Focal Loss (FL)	Adam optimizer, learning rate = 0.001, batch size = 32, focal loss for class imbalance	89.48%	
^26^		Contrast enhancement	No	DarkNet-53 and DenseNet-201 using transfer learning	-Epochs = 100-Learning rate = 0.0002,-Momentum = 0.6557-Batch size = 128	85.4%	
^37^		Data augmentation and resize	No	Inception ResNet v2 and EfcientNet-B4 ensemble	Adam optimizer with lr = 0.01, Epsilon = 0.1	88.21%	
^38^		Resize, data augmentation and normalization	No	ConvNext-Tiny, EfficientNetB0, SENet, DenseNet, ResNet50 ensemble	Adam optimizer with lr = 0.001-Epochs = 100-Batch size = 32	90.15%	
^39^		Image resizing	No	The DXDSENet-CM ensemble model combines Xception, DenseNet201, and a Depthwise Squeeze-and-Excitation ConvMixer for enhanced skin lesion classification	Adam optimizer with learning rate schedule ReduceLROnPlateau (factor 0.3, min_lr 1e-6),Batch size: 128 Epochs: 100 Input size: 224 × 224 Activation:GELU ReLU, Softmax	88.21%	
^28^		Image resizing -Normalization	No	Federated MobileNetV2	4 clients total (2 trained on ISIC 2018); 7 classes	80%	
^29^	ISIC 2019	Image: Data augmentation resizingMetadata: Standardization Missing metadata (mean imputation for numerical values and mode imputation for categorical values)	Yes	Ensemble of EfficientNet models (EfficientNetB0, EfficientNet-B1, EfficientNet-B2) for image path, metadata processed through separate path and fused with image features for final classification	Adam optimizer with lr = 0.001, batch size = 32	74.2% (balanced accuracy)	**91.1%**
^30^		Data augmentation, colour constancy (Shades of Gray), metadata encoding	Yes	EfficientNet-B3 & B4 with metadata fusion, TTA	SGD, OneCycle LR, weighted cross-entropy loss	66.2%	
^35^		Black borders removal andreal time data augmentation	No	Ensemble of EfficientNet-B5, SE-ResNeXt-101(32 × 4d),EfficientNet-B4 andInception-ResNet-v2	-Number of epochs over 32- Weighted Cross-Entropy Loss	63.4%	
^36^		Normalization, data augmentation and cropping	No	Ensemble of DenseNet-V2,Inception-V3,InceptionResNetV2 andXception	-Adam optimizer with learning rate (initial) = 1e-3Learning rate = 1e-4-Epochs = 50 (starting from the 4th epoch-Batch size = 64	82.1%	
^32^		Image: -Metadata: Clinical data (age, sex, medical history) integrated	Yes	DenseNet-169 with MetaNet and MetaBlock modules	Adam optimizer, learning rate = 0.001, batch size = 32	81.4% balanced accuracy	
^40^		-Image resizing (100 × 100),-Non-Local Means denoising,-Data augmentation	No	Custom CNN with Sparse Dictionary Learning	Adam optimizer, batch size = 32, epochs = 100, filters (128, 256, 512, 512, 256), kernel sizes (11 × 11 → 1 × 1), ReLU and Softmax activations	81.23%	
^27^		-image resizing (299 × 299)-Data augmentation -Class balancing through oversampling	No	Inception-V3	Adam optimizer, learning rate = 0.01, dropout = 0.25, batch size = 20, epochs = 50, fivefold cross-validation	88.63%	
^28^		Image resizing -Normalization	No	Federated MobileNetV2	4 clients total (2 trained on ISIC 2019); 8 classes	87%	

Significant values are in [bold].

Table 17 presents the summary of comparing the performance of the proposed model on the ISIC 2018 and 2019 datasets against various related works. First, for the ISIC 2018 dataset, the proposed model demonstrates significant improvements in accuracy.

For ISIC 2018, compared to^30^, which reported 89.5%, the proposed model surpasses it by 4.13%. Their study incorporated metadata fusion with EfficientNet‑B3 and B4, test‑time augmentation (TTA), and colour constancy pre‑processing. Although metadata fusion contributed to their model’s improved performance, dataset complexity and class imbalance constrained further accuracy gains.

Similarly, compared to^31^, which achieved 89.48%, the proposed model shows a 4.15% improvement. This study leveraged a hybrid CNN‑ViT architecture with Focal Loss (FL) and metadata handling techniques such as normalization and distribution balancing. While metadata integration improved classification performance, our enhanced feature extraction and balancing strategies resulted in superior accuracy.

Compared to^26^, which achieved 85.4%, the proposed model demonstrates an improvement of 9.13%. Their method applied contrast enhancement as a pre‑processing step and used DarkNet‑53 and DenseNet‑201 with transfer learning. While contrast enhancement enhances lesion visibility, it lacks the comprehensive feature extraction capabilities integrated into our approach.

In comparison to^37^, which achieved 88.21%, the proposed model shows a 5.66% improvement. Their study utilized an ensemble of Inception ResNet v2 and EfficientNet‑B4 trained with an Adam optimizer. While their model leveraged strong architectures, the absence of metadata utilization limited its ability to incorporate additional clinical features for enhanced classification.

The proposed model also shows a 3.38% improvement over^38^, which obtained 90.15%. This study employed an ensemble of ConvNext‑Tiny, EfficientNetB0, SENet, DenseNet, and ResNet50 with pre‑processing techniques such as resizing, data augmentation, and normalization. Although their diverse ensemble approach contributed to robust feature extraction, our optimized model integrates advanced learning strategies that improve classification accuracy.

Recent studies^39^ have shown that ensemble and attention‑based deep learning frameworks significantly enhance skin lesion classification. For example, DXDSENet‑CM (2024) combined Xception, DenseNet201, and a Depthwise Squeeze‑and‑Excitation ConvMixer (DSENet‑ConvMixer) to leverage the strengths of multiple convolutional backbones and attention mechanisms. This ensemble achieved an accuracy of 88.21% on the ISIC 2018 dataset; compared to this, the proposed ensemble model shows a 5.66% improvement, demonstrating superior ability to improve classification performance and generalization across diverse dermoscopic images.

Lastly, compared to^28^, which achieved 80% accuracy on ISIC 2018, the proposed ensemble model demonstrates a 16.5% improvement. Their study leveraged a federated learning framework combining CNN and MobileNetV2 models to ensure data privacy while addressing domain shifts across distributed datasets. While federated learning enhanced cross‑dataset adaptability and privacy preservation, our ensemble’s advanced feature extraction and model integration strategies contributed to significantly higher classification accuracy.

Regarding the ISIC 2019 dataset, we compared to^29^, which obtained 74.2%, the proposed model shows a 22.7% improvement. Their ensemble of EfficientNet models (B0, B1, B2) applied image‑based data augmentation and standardization, with metadata handled via imputation, but the proposed model’s advanced feature extraction strategies led to higher performance.

Similarly, we compared the performance of the proposed model with several related works, also showing substantial improvements in accuracy. Compared to^30^, which reported 66.2%, the proposed model surpasses it by 37.61%. This study integrated EfficientNet‑B3 and B4 with metadata fusion, but the proposed model’s optimization strategies and refined metadata utilization led to superior accuracy.

Compared to^35^, which achieved 63.4%, the proposed model shows an improvement of 43.7%. Their ensemble approach included EfficientNet‑B5 and SE‑ResNeXt‑101, but their model lacked metadata integration, which limited its accuracy.

Compared to^36^, which achieved 82.1%, the proposed model demonstrates a 10.96% improvement. Their study used an ensemble of DenseNet‑V2, Inception‑V3, and Xception with pre‑processing techniques like normalization and data augmentation, but the proposed model’s advanced learning strategies and metadata integration contributed to its higher accuracy.

Compared to^32^, which achieved 81.4%, the proposed model outperforms it by 11.92%. Their study used DenseNet‑169 with MetaNet and MetaBlock modules but did not leverage metadata for enhanced classification.

In comparison to^40^, which achieved an accuracy of 81.23%, the proposed model shows a 12.16% improvement. Their method involved a custom CNN architecture combined with Sparse Dictionary Learning and pre‑processing steps such as image resizing (100 × 100), Non‑Local Means denoising, and data augmentation. While effective, the significant performance gap can be attributed to the proposed model’s use of deeper ensemble architectures, advanced feature integration, and inclusion of clinical metadata, which collectively enhance its generalization and decision‑making capabilities.

Lastly, in^27^, which achieved an accuracy of 88.63%, the proposed model demonstrates a 2.78% improvement. Their approach utilized the Inception‑V3 architecture along with pre‑processing techniques such as image resizing (299 × 299), data augmentation, and class balancing through oversampling. Despite their use of a strong baseline and cross‑validation, the proposed model’s integration of metadata and ensemble learning further enhances classification performance, leading to a more robust and accurate system for skin lesion analysis.

## Conclusion and future work

This study fundamentally advances the domain of automated skin cancer detection by demonstrating the critical synergy between metadata integration, sophisticated pre-processing, and adaptive ensemble learning. Beyond improving raw classification metrics, this study collectively addresses key challenges such as data imbalance, variability in clinical metadata, and the heterogeneity of dermoscopic images, thereby pushing the limits of CNN applicability in real-world medical contexts.

The adaptive weighted ensemble approach—combining ResNet50, Xception, and EfficientNetB0—exemplifies how dynamically optimized model fusion can outperform conventional fixed-weight or simple averaging ensembles, resulting in enhanced robustness and generalization across multiple datasets. The proposed model achieved 93.2% accuracy, 93% precision, 93% recall, 93% F1 score, and 97.3% AUC on ISIC 2018. On ISIC 2019, it reached 91.1% accuracy, 92% precision, 93% recall, 92% F1 score, and 95.5% AUC. On the external Derm7pt dataset, without fine-tuning, the model maintained strong performance with 82.5% accuracy, 86% precision, 83% recall, 84% F1 score, and 89.15% AUC, demonstrating strong and balanced performance even under domain shift conditions. The strategic incorporation of metadata not only enriches feature representation but also facilitates more nuanced decision boundaries, highlighting the untapped potential of multimodal data in dermatological diagnostics.

Future work could focus on how metadata contributes to individual predictions and its impact on refining the fusion process, leading to better generalization across different datasets. These improvements can further strengthen the reliability and clinical relevance of automated skin cancer detection systems.

## Data Availability

The ISIC 2018 and ISIC 2019 datasets analysed during the current study are available in the ISIC archive repository, https://challenge.isic-archive.com/ Additionally, the Derm7pt dataset used in this study is available on Kaggle at https://www.kaggle.com/datasets/menakamohanakumar/derm7pt
